# Mechanistic insight into activation of MAPK signaling by pro-angiogenic factors

**DOI:** 10.1186/s12918-018-0668-5

**Published:** 2018-12-27

**Authors:** Min Song, Stacey D. Finley

**Affiliations:** 1 Department of Biomedical Engineering, University of Southern California, Los Angeles, California USA; 2 Department of Chemical Engineering and Materials Science, University of Southern California, Los Angeles, California USA; 3 Department of Biological Sciences, Computational Biology section, University of Southern California, 1042 Downey Way, CRB 140, Los Angeles, CA 90089 USA

## Abstract

**Background:**

Angiogenesis is important in physiological and pathological conditions, as blood vessels provide nutrients and oxygen needed for tissue growth and survival. Therefore, targeting angiogenesis is a prominent strategy in both tissue engineering and cancer treatment. However, not all of the approaches to promote or inhibit angiogenesis lead to successful outcomes. Angiogenesis-based therapies primarily target pro-angiogenic factors such as vascular endothelial growth factor-A (VEGF) or fibroblast growth factor (FGF) in isolation. However, pre-clinical and clinical evidence shows these therapies often have limited effects. To improve therapeutic strategies, including targeting FGF and VEGF in combination, we need a quantitative understanding of the how the promoters combine to stimulate angiogenesis.

**Results:**

In this study, we trained and validated a detailed mathematical model to quantitatively characterize the crosstalk of FGF and VEGF intracellular signaling. This signaling is initiated by FGF binding to the FGF receptor 1 (FGFR1) and heparan sulfate glycosaminoglycans (HSGAGs) or VEGF binding to VEGF receptor 2 (VEGFR2) to promote downstream signaling. The model focuses on FGF- and VEGF-induced mitogen-activated protein kinase (MAPK) signaling and phosphorylation of extracellular regulated kinase (ERK), which promotes cell proliferation. We apply the model to predict the dynamics of phosphorylated ERK (pERK) in response to the stimulation by FGF and VEGF individually and in combination. The model predicts that FGF and VEGF have differential effects on pERK. Additionally, since VEGFR2 upregulation has been observed in pathological conditions, we apply the model to investigate the effects of VEGFR2 density and trafficking parameters. The model predictions show that these parameters significantly influence the response to VEGF stimulation.

**Conclusions:**

The model agrees with experimental data and is a framework to synthesize and quantitatively explain experimental studies. Ultimately, the model provides mechanistic insight into FGF and VEGF interactions needed to identify potential targets for pro- or anti-angiogenic therapies.

**Electronic supplementary material:**

The online version of this article (10.1186/s12918-018-0668-5) contains supplementary material, which is available to authorized users.

## Background

Angiogenesis is the formation of new blood capillaries from pre-existing blood vessels. The essential role of blood vessels in delivering nutrients makes angiogenesis important in the survival of tissues, including tumor growth. Angiogenesis also provides a route for tumor metastasis. Thus, targeting angiogenesis is a prominent strategy in many contexts, for example, in both tissue engineering and cancer treatment.

In the context of tissue engineering, there is a large demand for organs needed for transplant surgery, but a great shortage of donors. The long-term viability of engineered tissue constructs depends on growth of new vessels from host tissue, and stimulating new blood vessel formation is an important pro-angiogenic strategy for tissue engineering [1]. Alternatively, the formation of new blood vessels is important for cancer growth and metastasis. Thus, inhibiting angiogenesis is an anti-angiogenic strategy for cancer treatment. Unfortunately, not all approaches to promote or inhibit angiogenesis lead to successful outcomes. For example, clinical trials have shown no effective improvement in blood flow or perfusion by fibroblast growth factor (FGF)-induced [2] or vascular endothelial growth factor-A (VEGF)-induced [3] angiogenesis. Specifically, a double-blinded randomized controlled trial studied recombinant FGF-induced angiogenesis and showed no symptomatic improvement (exercise tolerance or myocardial perfusion) following 90 or 180 days of treatment [2]. Similarly, in a double-blinded placebo-controlled trial to study the effects of recombinant human VEGF-induced angiogenesis in animal models, there was no improvement in angina, in comparison with placebo by day 60. Only a high dose of VEGF (50 ng/kg/min) showed any effect [3]. Also, bevacizumab, an anti-VEGF agent for cancer treatment, has limited effects in certain cancer types, and it is no longer approved for the treatment of metastatic breast cancer due to its disappointing results [4]. Thus, there is a need to better understand the molecular interactions and signaling required for new blood vessel formation, in order to establish more effective therapeutic strategies.

The established angiogenesis-based therapies primarily target pro-angiogenic factors such as FGF and VEGF in isolation. However, both FGF and VEGF bind to their receptors to initiate mitogen-activated protein kinase (MAPK) signaling and phosphorylate ERK, the final output of the MAPK pathway [5, 6]. This signaling pathway promotes cell proliferation in the early stages of angiogenesis. Additionally, the combined effects of FGF and VEGF have been shown to be greater than their individual effects [7, 8]. A quantitative understanding of how these promoters combine together to stimulate angiogenesis could greatly benefit the current pro- and anti-angiogenic therapies.

Mathematical modeling is a useful tool to predict the molecular response mediated by angiogenic factors. For example, Mac Gabhann and Popel studied interactions between VEGF isoforms, VEGF receptors (VEGFR1, VEGFR2, NRP1), and the extracellular matrix using a molecular-detailed model. The model predicted that blocking Neuropilin-VEGFR coupling is more effective in reducing VEGF-VEGFR2 signaling than blocking Neuropilin-1 expression or binding of VEGF to Neuropilin-1 [9]. Stefanini et al. constructed a pharmacokinetic model that studied VEGF distribution after intravenous administration of bevacizumab, and they found that plasma VEGF was increased after treatment [10]. Filion and Popel explored myocardial deposition and retention of FGF after intracoronary administration of FGF using a computational model. The model predicted that the response time is dependent on the reaction time of the binding of FGF to FGFR rather than the FGF diffusion time. Receptor secretion and internalization have also been predicted to be crucial in FGF dynamics [11]. Wu and Finley characterized the intracellular signaling of TSP1-induced apoptosis and predicted responses of cell populations to TSP1-mediated apoptosis by mathematical modeling [12]. Zheng et al. integrated the effects of VEGF, angiopoietins (Ang1 and Ang2) and platelet-derived growth factor-B (PDGF-B) on endothelial proliferation, migration, and maturation using mathematical modeling. Their model illustrated that competition between Ang1 and Ang 2 acts as an angiogenic switch and that combining anti-pericyte and anti-VEGF therapy is more effective than anti-VEGF therapy alone in inducing blood vessel regression [13]. Such models are useful to predict molecular responses of VEGF or FGF stimulation; however, surprisingly, the interactions between these two growth factors have not been investigated in detail. Targeting multiple growth factors simultaneously, exploiting their overlapping and redundant signaling pathways, may improve angiogenesis-based therapies. Thus, there is a need for a model that provides quantitative insights into combination effects of FGF and VEGF.

In the present study, we aim to quantitatively characterize the crosstalk between FGF and VEGF in MAPK signaling leading to phosphorylated ERK (pERK). We focus on pERK because this species promotes cell proliferation [14] and is mostly found in active, rather than quiescent, endothelial cells [15]. We constructed a computational model that incorporates the molecular interactions between FGF, VEGF, and their receptors, leading to MAPK signaling. We apply the model to explore how FGF and VEGF promote ERK phosphorylation. This is the first model that studies FGF and VEGF interactions together on a molecular level. Our model predicts the combination effects of FGF and VEGF stimulation and shows that FGF plays a dominant role in promoting ERK phosphorylation. Using this model, we also investigated the effects of the VEGF receptor VEGFR2, including how VEGFR2 density and trafficking parameters influence the ERK response. By predicting the effect of VEGFR2 density and trafficking parameters, we can get a better understanding of the role of VEGFR2 under pathological conditions. Additionally, understanding with quantitative detail the FGF and VEGF interactions helps identify potential targets for enhancing pro- or anti-angiogenic therapies.

## Results

### The validated mathematical model captures the main features of FGF- and VEGF-stimulated ERK phosphorylation dynamics

**Fig. 1:**
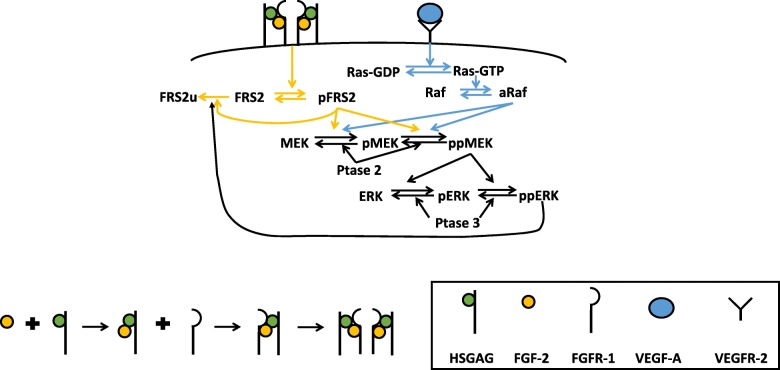
Schematic of FGF and VEGF signaling network. Signaling is induced by the growth factors binding to their receptors, culminating with phosphorylation of ERK, through the MAPK cascade. MAPK signaling is initiated through the activation of Raf and FRS2 by VEGF and FGF, respectively. The FGF:HSGAG:FGFR1 complex dimerizes and leads to phosphorylation of FRS2 (pFRS2). VEGF binds VEGFR2 to activate Ras, forming Ras-GTP, which further activates Raf (aRaf). Both aRaf and pFRS2 are able to phosphorylate MEK at two sites, and doubly phosphorylated MEK (ppMEK) further phosphorylates ERK. ppERK provides negative feedback on the FGF pathway, as it promotes ubiquitination of FRS2 (FRS2u)

We constructed a computational model that characterizes FGF and VEGF interactions leading to ERK phosphorylation (Fig. 1). Signaling is mediated by FGF and VEGF binding to their respective receptors, leading to pERK. The model was trained against published experimental measurements [16–18] using Particle Swarm Optimization (PSO) [19] for parameter estimation. We note that pERK response stimulated by FGF was measured using the non-small cell lung cancer cell line NCI-H1730 [16], while phosphorylated VEGFR2 (pR2) [18] and pERK [17] responses induced by VEGF were obtained using human umbilical vein endothelial cells (HUVECs). In this study, we assume the downstream signaling has the same kinetics across different cell lines. The FGFR1 and heparan sulfate glycosaminoglycan (HSGAG) levels on various cell types are fairly consistent: approximately 10^4^ to 10^5^ molecules/cell for Balb/c3T3 [20], osteoblasts and bone marrow stromal cells [21], bovine aortic endothelial cells (BAECs) [22], and the NCI-H1730 cell line [16]. In addition, other models have made similar assumptions that signaling is consistent across cell types, for example, using HUVEC data to study macrophage signaling responses [23]. That work, which supposes that the unique signaling responses for specific cell types are due to their different protein levels rather than the kinetics, is able to match experimental data. Thus, we assume the FGFR1 signaling pathway for NCI-H1730 cells is the same as in endothelial cells.

To explore the behavior of the model, we performed a global sensitivity analysis, which identifies the variables that significantly influence the model outputs. Specifically, we performed the extended Fourier Amplitude Sensitivity Test (eFAST) (see Methods for more details) and computed the total sensitivity indices (*S*_ti_) for initial conditions or kinetic parameters on pERK level by the stimulation of FGF or VEGF. The eFAST results reveal the specific species and kinetic parameters that affect pERK. These results show the importance of particular species (Additional file 1: Figure S1A): FRS2 and Ptase2 for FGF-induced signaling and Ras, MEK, and Ptase2 for VEGF-induced signaling. Additionally, the rates of certain reactions involving these species are also shown to be important (Additional file 1: Figure S1B). This includes “*ked2*” and “*k_dpMEK_p*”, which are the dephosphorylation rate of ppMEK by Ptase2 and the association rate of pMEK and Ptase2, respectively. Moreover, the eFAST results indicate the importance of the VEGFR2 trafficking parameters. Specifically, the internalization rates of bound VEGFR (“*k_intb*”, “*k_recb*”, and “*k_degb*”) are shown to affect ppERK with VEGF stimulation (Additional file 1: Figure S1B, bottom two panels). These results provide the foundation to investigate how the VEGFR2 trafficking rates influence pERK dynamics, which we explore below. In addition, these results can guide parameter fitting. Since many of the influential parameters and initial values are involved in the overlap of FGF and VEGF signaling pathways, along with the VEGFR2 trafficking parameters, we selected those values for model fitting.

**Fig. 2:**
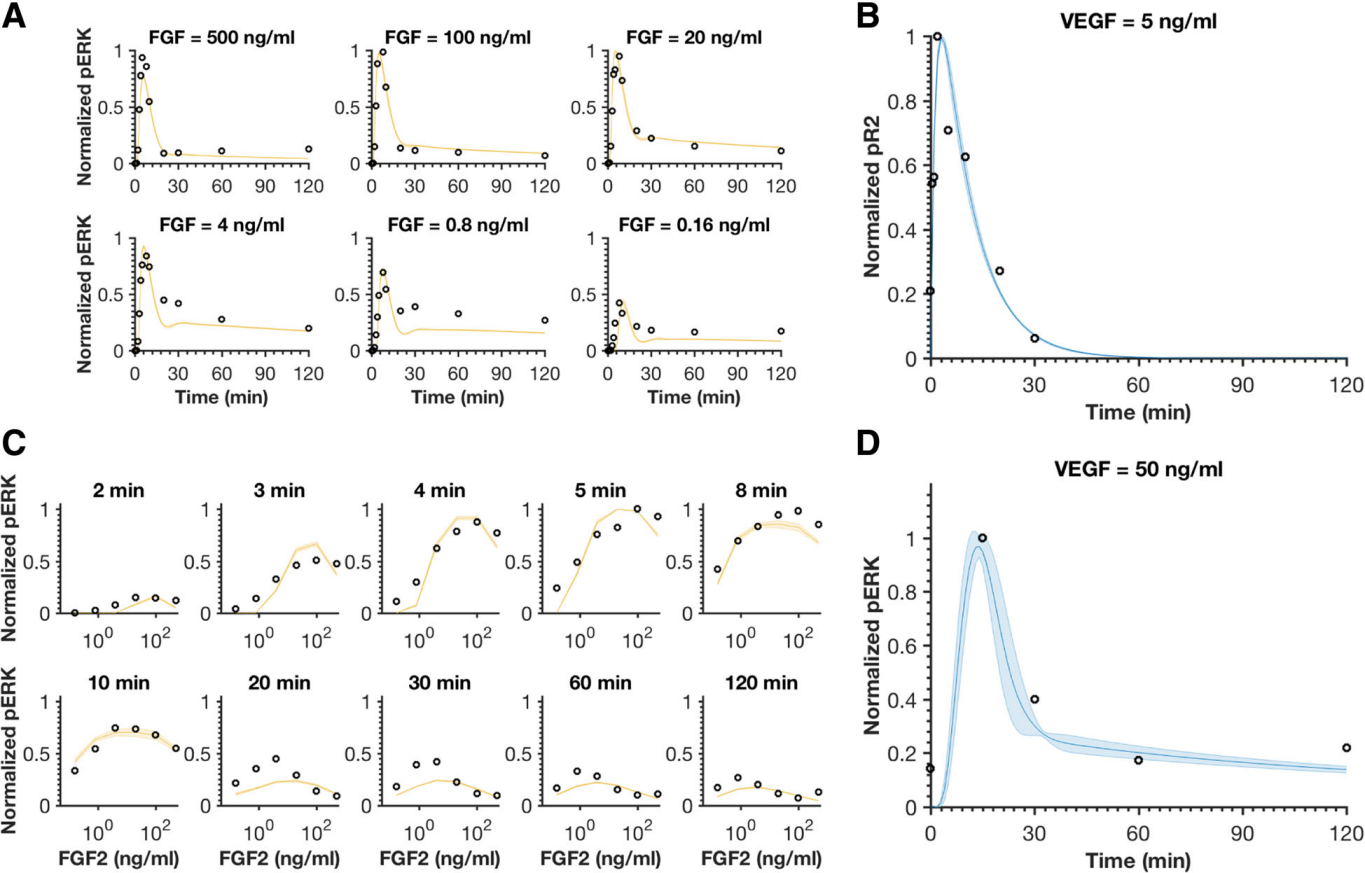
Model comparison to training data for FGF or VEGF stimulation. **a** Normalized pERK dynamics in response to FGF concentrations ranging from 0.16 to 500 ng/ml. **b** Normalized VEGFR2 phosphorylation time course following stimulation with 5 ng/ml VEGF. **c** Dose response of pERK for FGF stimulation. **d** Normalized ERK phosphorylation time course upon stimulation with 50 ng/ml VEGF. The circles are experimental data. Curves are the mean values of the 16 best fits. Shaded regions show standard deviation of the fits

The fitted model shows a good match to the training data (Fig. 2). It can capture the biphasic pERK response caused by FGF stimulation, which has been reported by Kanodia et al. (Fig. 2a, c): pERK increases as the FGF concentration increases from low to intermediate levels, and decreases with increasing FGF at high concentrations. The decrease in pERK is caused by the competitive binding of FGF to HSGAG and FGFR [16]. At lower FGF levels, there are enough FGFR molecules to bind with FGF, forming FGF-FGFR complexes that can interact with HSGAGs to form the pro-angiogenic tertiary complex (FGF-HSGAG-FGFR). However, at higher FGF levels, formation of the FGF-FGFR complex is limited by the number of FGFR molecules, which thus limits the formation of the tertiary complex. This is primarily due to the different expression levels of FGFR and HSGAGs (2 × 10^4^ versus 10^5^ molecules/cell, respectively) [16]. Our fitted model recapitulates this biphasic response at the simulated time points. Also, VEGF-induced upstream (pVEGFR2) and downstream (pERK) dynamics have good agreement with experimental measurements (Fig. 2b, d) [17, 18]. For the best 16 fits, the weighted errors range from 17.4 to 18.6 (Additional file 2: Table S1). In addition, the fitted parameters have good consistency. We allowed the values of the initial conditions and kinetic parameters that were fitted to vary up to two orders of magnitude (10-fold above and below the baseline starting value). We examined the results of the 16 best parameter sets, focusing on the range spanning two standard deviations above and below the mean for each fitted initial condition and parameter. For all the fitted initial conditions and 32 of the 34 fitted parameters, this range was within one order of magnitude (Additional file 1: Figure S2). Thus, the fitting reduced the bounds.

**Fig. 3:**
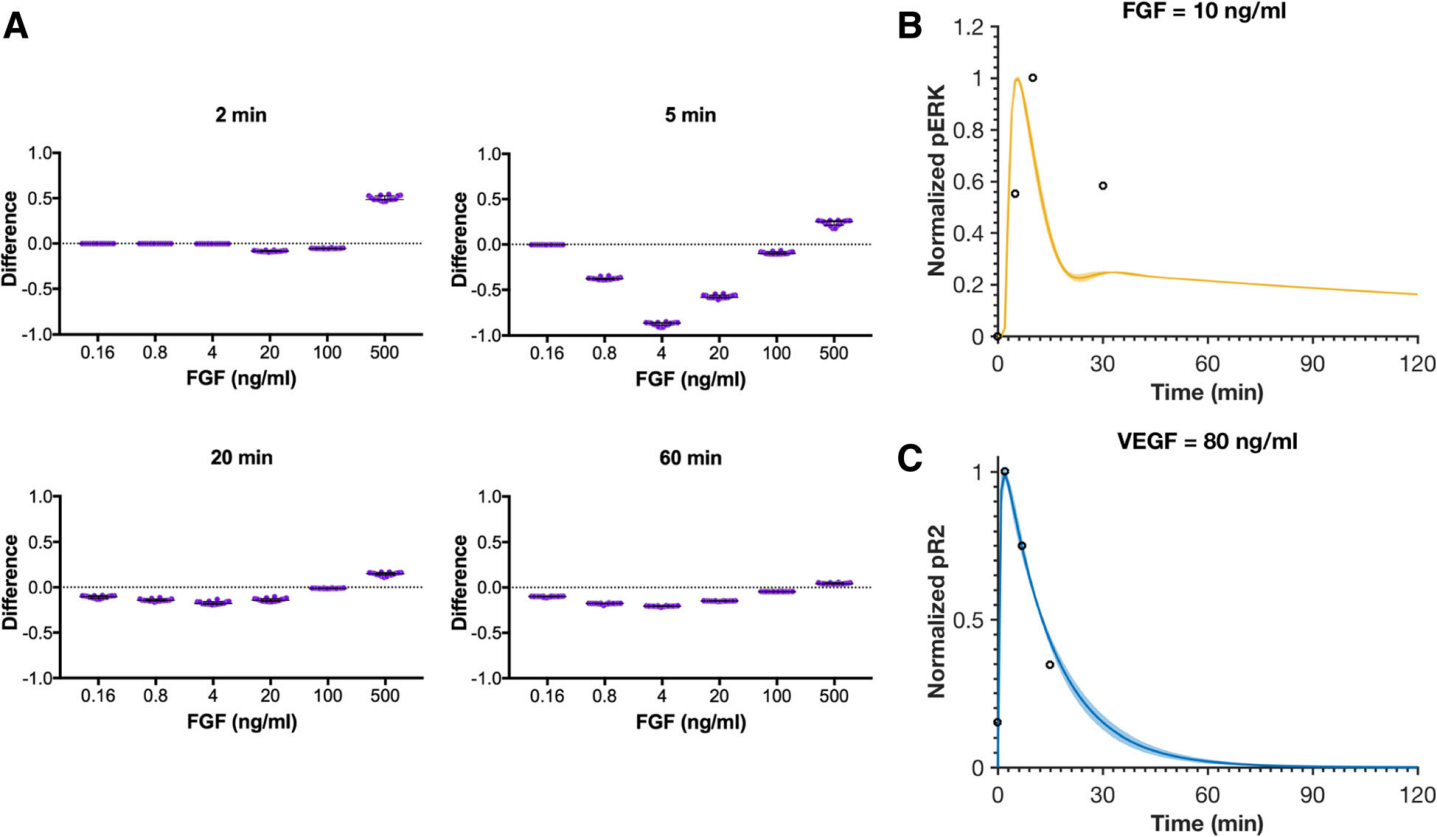
Model comparison to validation data. **a** The differences in pERK induced by stimulation of FGF with and without 500 μg/ml heparin at four time points are predicted. Each dot represents one fit. The dots are spread horizontally to avoid overlap of similar responses from different fits. **b** Normalized pERK by the stimulation of 10 ng/ml FGF. Circles are BAEC experimental data. **c** Normalized VEGFR2 phosphorylation time course upon stimulation with 80 ng/ml VEGF. Circles are HUVEC experimental data. Curves are the mean values of the 16 best fits from the model training. Shaded regions show standard deviation of the fits

To validate the model, we compared the predictions to additional experimental data. We first applied heparin perturbation to the trained model to reproduce another set of data by Kanodia et al. Heparin is a soluble source of HSGAGs, and it competes with HSGAGs to bind with FGF, interfering with FGF-induced signaling (Additional file 1: Figure S3) [16]. It has been reported that additional heparin increases FGF-induced ERK phosphorylation at high FGF concentrations, and decreases FGF-induced ERK phosphorylation at low FGF concentrations in two hours [16]. We validated the model by expanding it to include heparin binding and comparing to the experimental measurements. We added 500 μg/ml of heparin, the same concentration used in experiments [16], and found that the difference of predicted pERK responses upon FGF stimulation with and without heparin within two hours exhibits the trend observed to occur experimentally: at high FGF concentrations, the difference between the presence and absence of heparin is greater than zero, while the difference is less than zero at lower FGF concentrations (Fig. 3a). This shows a qualitative agreement with experimental observations.

A separate set of experimental measurements of phosphorylated ERK by the stimulation of 10 ng/ml FGF conducted using BAECs [24] was extracted to further validate FGF-induced endothelial signaling. Our model also has a good agreement with this experimental data (Fig. 3b), which further confirms that this model can be used to predict endothelial cell signaling.

Finally, we extracted an independent set of experimental measurements of the phosphorylated VEGFR2 response by the stimulation with 80 ng/ml VEGF in HUVECs [25] to validate the VEGF-induced signaling. Our model quantitatively matches the experimental data (Fig. 3c). Overall, we find that our trained model can generate reliable predictions for the endothelial intracellular signaling response stimulated by FGF or VEGF. This further supports our assumption that different data sets can be used to establish a predictive model of signaling in ECs. Thus, we used the best fits (based on model training and validation) in subsequent predictions and analyses.

### FGF produces a greater angiogenic response than VEGF when considering the maximum ERK phosphorylation produced

Using the validated model, we first studied the effects of FGF and VEGF individually on pERK. We simulate a range of FGF and VEGF concentrations, based on several experimental studies published in literature (see Methods). Specifically, we chose the ligand concentration range of 0.01–2 nM to investigate the ERK activation in response to typical levels of FGF and VEGF in in vitro studies.

**Fig. 4:**
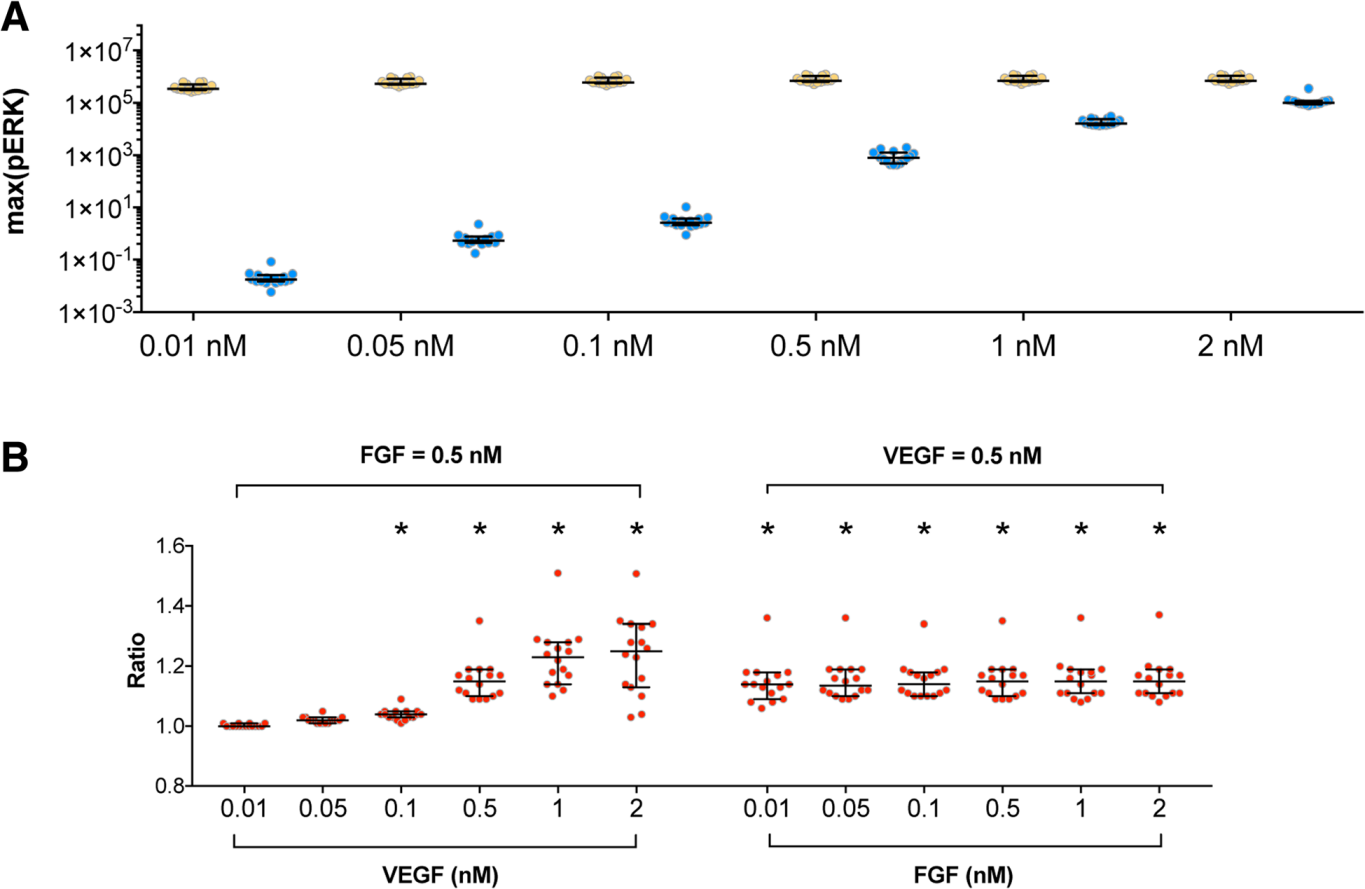
Predicted maximum pERK response. **a** Maximum pERK in response to FGF (yellow) or VEGF (blue) concentrations varying from 0.01 nM to 2 nM. **b** Ratio, *R*, of combination effects to the summation of individual effects in response to FGF and VEGF. Each dot represents one fit. The dots are spread horizontally to avoid overlap of similar responses from different fits. Asterisk indicates statistically significant difference compared with one (*p* < 0.05). Bars are median ± 95% confidence interval

The model predicts that FGF is more potent in promoting ERK phosphorylation, compared to VEGF, at equimolar concentrations. When FGF concentration is varied from 0.01 nM to 2 nM, the maximum pERK ranges from 4 × 10^5^ molecules/cell to 8 × 10^5^ molecules/cell, while VEGF induces a maximum of 2 × 10^− 2^ molecules/cell to 1 × 10^5^ molecules/cell pERK for the same concentration range (Fig. 4a). For example, on average (across the 16 best fits), the maximum pERK induced by 0.5 nM FGF is 8 × 10^5^ molecules/cell, while 0.5 nM VEGF induces a maximum pERK of 9 × 10^2^ molecules/cell. Thus, FGF produces a maximum ERK phosphorylation that is approximately three orders of magnitude higher than that induced by VEGF. Furthermore, the maximum pERK is more sensitive to increasing the VEGF concentration, as compared to FGF. The maximum pERK increases steadily with increasing VEGF stimulation, while maximum pERK remains relatively constant as the level of FGF stimulation increases. The measurements from Kanodia et al. show that FGF stimulation does not significantly change the maximal pERK level (Fig. 2a) for concentrations ranging from 0.8 ng/ml (0.03 nM) to 100 ng/ml (4 nM) [16]. This experimental observation agrees with our model predictions for FGF stimulation shown in Fig. 4a. We can explain this result by examining the levels of the intermediate signaling species. For the FGF pathway, the phosphorylated trimeric complex of FGF, FGFR, and HSGAG binds to and phosphorylates FRS2, and phosphorylated FRS2 (pFRS2) leads to the phosphorylation of MEK. The resulting doubly phosphorylated MEK (ppMEK) further mediates the phosphorylation of ERK (Fig. 1). We found that even with 0.01 nM FGF stimulation, FRS2 is rapidly depleted (Additional file 1: Figure S4A). The shortage of FRS2 limits ppMEK level, which is the substrate for ERK phosphorylation and further limits pERK level. Therefore, FRS2 level limits the FGF-induced ERK phosphorylation. Increasing FGF concentration 200-fold (from 0.01 nM to 2 nM) only doubles the maximum pERK (from 4 × 10^5^ molecules/cell to 8 × 10^5^ molecules/cell), again due to the shortage of FRS2. The importance of FRS2 is also shown in the sensitivity analysis (Additional file 1: Figure S1).

On the other hand, for the VEGF pathway, phosphorylated VEGFR2 produces Ras-GTP, which activates Raf. The activated Raf (aRaf) mediates phosphorylation of MEK. As in the FGFR pathway, ppMEK mediates ERK phosphorylation (Fig. 1). The model predicts that there is enough Raf and MEK supply even upon stimulation with a high concentration of VEGF (2 nM), as shown in Additional file 1: Figure S4B. Thus, the maximum pERK increases significantly with increasing VEGF concentration, compared with FGF-induced ERK phosphorylation. Furthermore, the maximum pERK induced by FGF or VEGF gets closer as VEGF concentration increases (Fig. 4a).

The model predicts that one of the main reasons why FGF induces a greater maximum pERK response compared to VEGF is related to differences at the receptor level. Despite depletion of FRS2, high FGFR levels enable robust FGF-mediated signaling. FGFR density is much higher than VEGFR2 density (20,000 molecules/cell compared to 1000 molecules/cell). Additionally, the trafficking parameters (internalization, recycling, and degradation rates) for FGFR are lower than the corresponding VEGFR2 trafficking parameters (Additional file 1: Figure S5). Although internalized VEGFR2 molecules are recycled back to the surface more rapidly than FGFR molecules, VEGFR2 is internalized and degraded more rapidly than FGFR. Additionally, the dynamics of FGF receptors and VEGFR2 in their signaling, internalized, and degraded forms upon stimulation with 0.5 nM FGF or 0.5 nM VEGF (Additional file 1: Figure S6) indicate that more FGFR is available to signal instead of being internalized or degraded (non-signaling), compared to VEGFR2.

### The combination of FGF and VEGF has greater effects in inducing maximum pERK than the summation of the individual effects

We next studied the combination effects of FGF and VEGF in inducing maximum pERK. Here, we define a ratio comparing the combination effects to the individual effects. Specifically, this ratio is the maximum pERK obtained with co-stimulation of FGF and VEGF over the summation of the maximum pERK for FGF and VEGF stimulation individually (see Methods for more details). In Fig. 4b (left panel), 0.5 nM FGF in combination with intermediate to high VEGF concentrations (0.1–2 nM) can produce a significantly greater maximal pERK response than the summation of their individual effects, as indicated by the ratios being significantly greater than one (*p* < 0.05). The ratios for combinations of VEGF concentrations at 0.01 or 0.05 nM with 0.5 nM FGF are slightly greater than one; however those differences are not statistically significant. Stimulation with 0.5 nM VEGF in combination with a FGF concentration as low as 0.01 nM can exhibit greater combined effects than the summation of their individual effects. For these cases, the ratios are all significantly greater than one (Fig. 4b, right panel).

Co-stimulation compensates for the limitations observed when only one pathway is stimulated. The model predicts that although VEGF-mediated MEK phosphorylation is much lower than FGF at the equimolar concentrations (Additional file 1: Figure S7A, reactions R26 and R28 compared with Additional file 1: Figure S7B, reactions R35 and R378), there are sufficient levels of Raf available compared to FRS2 (Additional file 1: Figure S4) to promote MEK phosphorylation. Thus, VEGF co-stimulation provides a way to overcome the limitation of the FRS2 level. On the other hand, in comparison with VEGF stimulation alone, the presence of FGF in the co-stimulation provides a high level of pMEK because MEK gets phosphorylated by pFRS2 much faster than by aRaf (Additional file 1: Figure S7C, reactions R26 and R28 compared with reactions R35 and R37). Together, these results explain why the combination of FGF and VEGF has a greater effect on ERK phosphorylation than the summation of their individual effect.

The effects of FGF and VEGF co-stimulation are more sensitive to VEGF, as compared to FGF. That is, increasing the VEGF concentration increases the ratio, while the ratio does not change with varying FGF concentrations. Additionally, the combination of FGF with VEGF shows a more additive response at low VEGF concentrations (< 0.1 nM). At higher concentrations (> 0.1 nM), increasing VEGF concentration increases the ratio (Fig. 4b).

Overall, the model predictions show that combinations of FGF and VEGF produce more ERK phosphorylation, compared to their individual effects. Additionally, the model indicates that VEGF-induced maximum pERK is more sensitive to varying the ligand concentration than FGF-induced maximum pERK, both for stimulation with VEGF or FGF alone and for co-stimulation.

### The combination of FGF and VEGF shows a fast and sustained pERK response

#### The combination of FGF and VEGF exhibits a fast pERK response

**Fig. 5:**
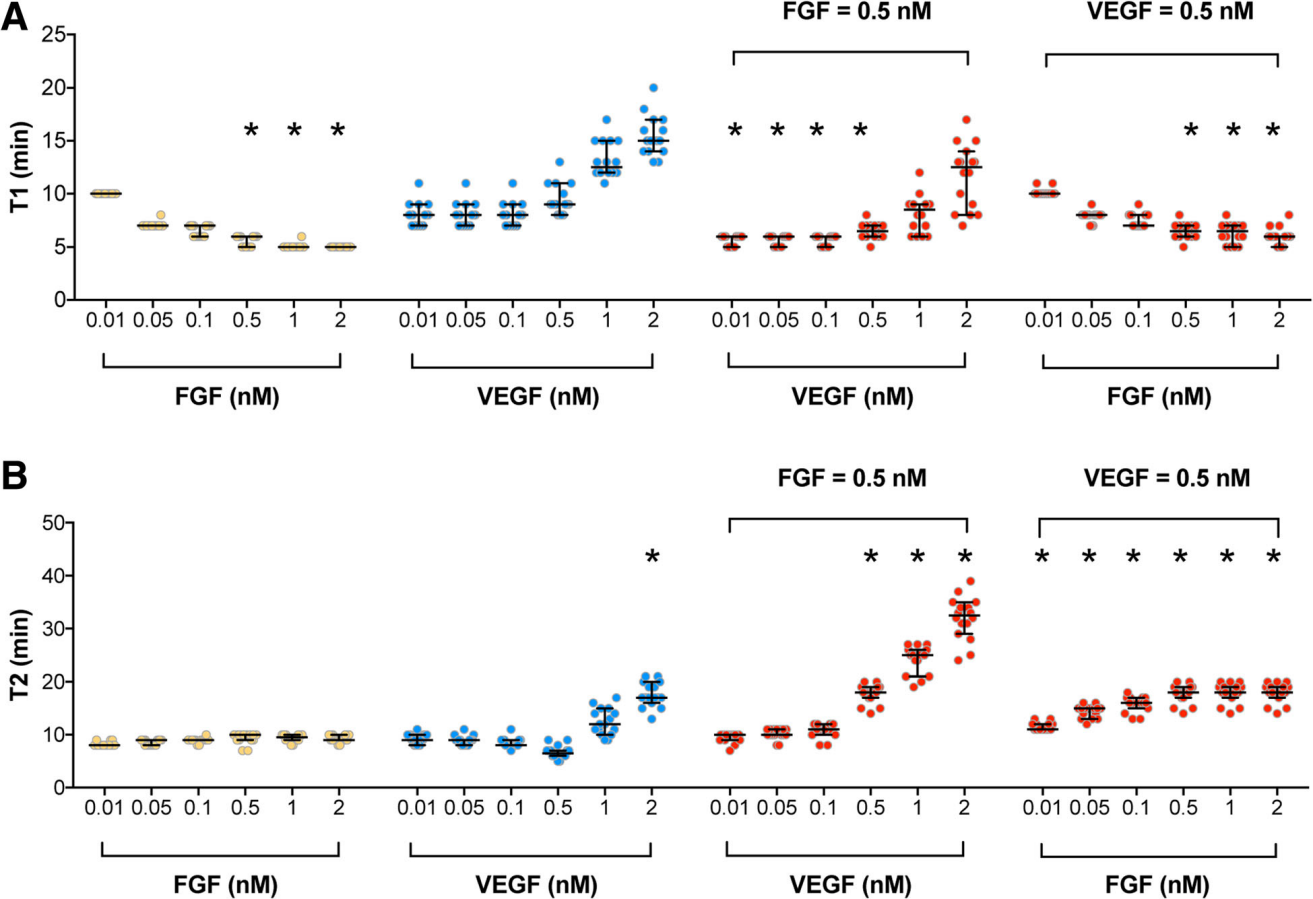
Predicted time response of pERK following stimulation by FGF, VEGF, and their combination. **a** *T1*, time to reach the maximum pERK in response to growth factor stimulation. Asterisk indicates statistically significant difference compared to corresponding VEGF concentration (*p* < 0.05). **b** *T2*, time that pERK is maintained above half of its maximum value in response to treatments. Each dot represents one fit. The dots are spread horizontally to avoid overlap of similar responses from different fits. Asterisk indicates statistically significant difference compared to corresponding FGF concentration (*p* < 0.05). Yellow: FGF; Blue: VEGF; Red: combination. Bars are median ± 95% confidence interval

In addition to studying the magnitude of the predicted pERK level upon FGF and VEGF mono- and co-stimulation, we investigated the timescale of the pERK response. First, we analyzed the time for the pERK level to reach its maximum value, termed “T1” (see Methods for more details) in response to the stimulation of FGF or VEGF individually. We found that FGF generally produces a faster response than VEGF stimulation at the same concentrations. Here, we characterize the timescale of the response in terms of the time it takes to reach maximum pERK. At low FGF concentrations (< 0.5 nM), the T1 values for FGF and VEGF are not significantly different (Fig. 5a). However, at high FGF concentrations (≥ 0.5 nM), FGF shows a significantly faster T1 response than VEGF. Specifically, for FGF concentrations ranging from 0.5 to 2 nM, the induced pERK response peaks within six minutes, while for the same range of VEGF concentrations, pERK reaches its peak value within 8 to 22 min (Fig. 5a). Experimental data from Kanodia et al. show that the values of T1 are all within eight minutes [16], and for 50 ng/ml (1.1 nM) VEGF stimulation, T1 is 15 min [17]. Thus, although we did not explicitly fit the model to the T1 values shown in experiments, our model predictions agree with those data. Together with Fig. 4a, the model predicts that FGF can induce a greater amount of ERK phosphorylation within a shorter period of time, compared to VEGF.

This difference in how T1 is affected by the two pro-angiogenic factors is caused by the availability of upstream species needed to activate MEK, and subsequently, ERK. As described above, on average, there is more Raf and aRaf available to promote downstream signaling upon VEGF stimulation, as compared to FRS2 and pFRS2 available with FGF stimulation. At low FGF concentrations, FRS2 is not depleted as quickly, and more ERK can be phosphorylated. As the FGF concentration used for stimulation increases, FRS2 is more rapidly depleted, and the maximal concentration of pERK happens more quickly. On the other hand, at high VEGF concentrations, there are still sufficient levels of Raf available to become phosphorylated and lead to ERK phosphorylation. Thus, the time to reach the maximal pERK concentration continues to increase as VEGF stimulation level increases.

As for the combination effects, we found that when the VEGF concentration is varied from 0.01 nM to 0.5 nM, co-stimulation with 0.5 nM FGF significantly speeds up ERK phosphorylation, compared to VEGF stimulation alone (Fig. 5a). For 0.5 nM VEGF stimulation, increasing the FGF concentration decreases T1, compared to VEGF stimulation alone. This decrease in T1 is significantly different than VEGF stimulation alone for FGF concentrations greater than 0.5 nM (Fig. 5a). Overall, these results indicate that pERK responds faster with FGF stimulation, as compared to VEGF stimulation.

#### The combination of FGF and VEGF induces sustained pERK response

We explored how long ERK can remain phosphorylated above its half-maximal value, termed “T2” (see Methods for more details), as another means of characterizing the timescale of the ERK response. The values of T2 for FGF and VEGF stimulation alone with concentrations ranging from 0.01 nM to 1 nM are not significantly different (T2 is approximately 9 min). However, a higher VEGF concentration produces a more sustained pERK response. Specifically, 2 nM VEGF shows significantly higher T2 (18 min on average) than 2 nM FGF stimulation (9 min on average) (Fig. 5b).

Regarding the combination effects, we found that with 0.5 nM FGF, VEGF concentrations greater than 0.5 nM are able to maintain ERK phosphorylation above its half-maximal value significantly longer, compared to FGF stimulation alone at the same concentrations. That is, T2 is significantly greater for combinations of 0.5 nM VEGF with FGF concentrations ranging from 0.01 nM to 2 nM, compared to FGF or VEGF stimulation alone (Fig. 5b).

To identify the reasons why pERK shows a more transient dynamic in response to FGF stimulation compared to VEGF stimulation at certain concentrations (> 0.5 nM), we compared the levels of the intermediate signaling species following stimulation of FGF or VEGF alone. For the co-stimulation of FGF and VEGF, the depletion of FRS2 still limits production of ppMEK with FGF stimulation (Additional file 1: Figure S8), similar to the case of mono-stimulation of FGF (Additional file 1: Figure S4). However, signaling through VEGFR compensates for this limitation (Additional file 1: Figure S8).

### Increasing VEGFR2 density can compensate for the relatively low ERK phosphorylation induced by VEGF

At its baseline level, the density of VEGFR2 is 20 times lower than FGFR1. This large difference contributes to the predicted results presented above. However, VEGFR2 upregulation has been observed in tumor growth. Experimental measurements of receptor expression show that some subpopulations of tumor endothelial cells (ECs) have high receptor levels: 13% of tumor-derived ECs have 7500 VEGFR2 molecules/cell after three weeks of tumor growth, and 5% of the tumor-derived ECs have 16,200 VEGFR2 molecules/cell after six weeks of tumor growth [26]. Therefore, we sought to understand the effects of varying the VEGF receptor density on the predicted pERK response to gain some insights into VEGF-mediated signaling in pathological conditions.

**Fig. 6:**
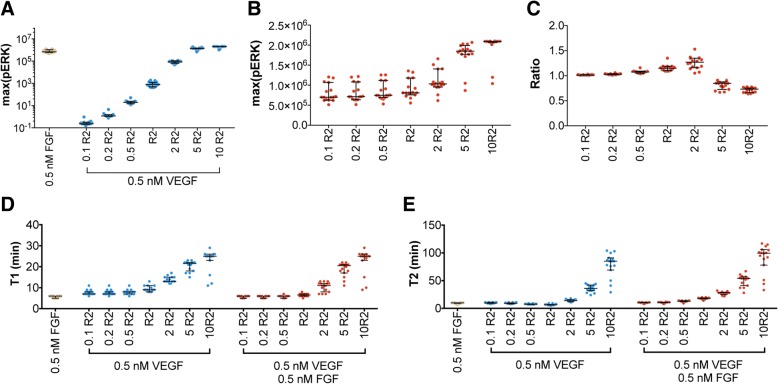
Predicted pERK response with varied initial VEGFR2 concentrations. **a** Maximum pERK induced by 0.5 nM FGF or 0.5 nM VEGF alone. **b** Maximum pERK induced by the combination of 0.5 nM FGF and 0.5 nM VEGF. **c** Ratio, *R*, of 0.5 nM FGF in combination with 0.5 nM VEGF. **d** *T1*, time to reach the maximum pERK in response to treatments. **e** *T2*, time that pERK is maintained above half of its maximum value in response to treatments. Yellow: FGF; Blue: VEGF; Red: combination. Each dot represents one fit. The dots are spread horizontally to avoid overlap of similar responses from different fits. Bars are median ± 95% confidence interval

We found that the maximum pERK induced by VEGF increases when VEGFR2 density increases (Fig. 6a). At equimolar concentrations of FGF and VEGF stimulation (0.5 nM), increasing VEGFR2 density by five-fold can increase the maximum pERK level to the same order of magnitude as FGF stimulation alone (Fig. 6b), which makes the effects of VEGF-mediated signaling sizeable in conditions of increased VEGFR2 density. Specifically, the model predicts that maximum pERK induced by the combination of FGF and VEGF increases more than 90% when VEGFR2 is increased by five-fold. In contrast, decreasing VEGFR2 by ten-fold leads to an 11.5% decrease in maximum pERK induced by the combination of FGF and VEGF. Thus, VEGFR2 density significantly impacts ERK phosphorylation with FGF and VEGF co-stimulation. In addition, the maximum ppERK level is higher upon stimulation by VEGF, compared to FGF, when VEGFR2 density is increased (Additional file 1: Figure S9A). Moreover, although the reaction rates for ERK phosphorylation by stimulation of FGF are slightly higher than VEGF during the first 10 min, VEGF induces higher rates between 10 to 60 min (Additional file 1: Figure S9B, reactions R42 and R43). In addition, VEGF exhibits faster phosphorylation for pERK than FGF (Additional file 1: Figure S9B, reactions R44 and R45). This indicates that the effect of VEGF is dominant in the combination effect when VEGFR2 density is increased by five-fold.

In Fig. 6c, as VEGFR2 density decreases, the ratio characterizing the ERK signaling response with a combination of FGF and VEGF compared to the summation of their individual effects becomes closer to one. This nearly additive combination effect occurs because reducing VEGFR2 makes the effect of VEGF stimulation negligible. Thus, the ratio is approximately one. The ratio increases when VEGFR2 density is increased by two-fold, which indicates a stronger combination effect. However, the summation of individual effects surpasses the combination effects (the ratio is less than one) when VEGFR2 density is more than five-fold higher than the baseline level. The reason for this is due to the competition between FGF and VEGF for downstream resources. Specifically, when VEGFR2 is increased more than two-fold, ERK is depleted (Additional file 1: Figure S10). This makes the combination effects less than the individual effects, causing the ratio to be less than one. Finally, by increasing VEGFR2, VEGF more strongly impacts the dynamics of pERK, as both T1 (Fig. 6d) and T2 (Fig. 6e) increase with increasing VEGFR2 density.

### ERK phosphorylation induced by VEGF can be promoted by decreasing VEGFR2 internalization and degradation rates

**Fig. 7:**
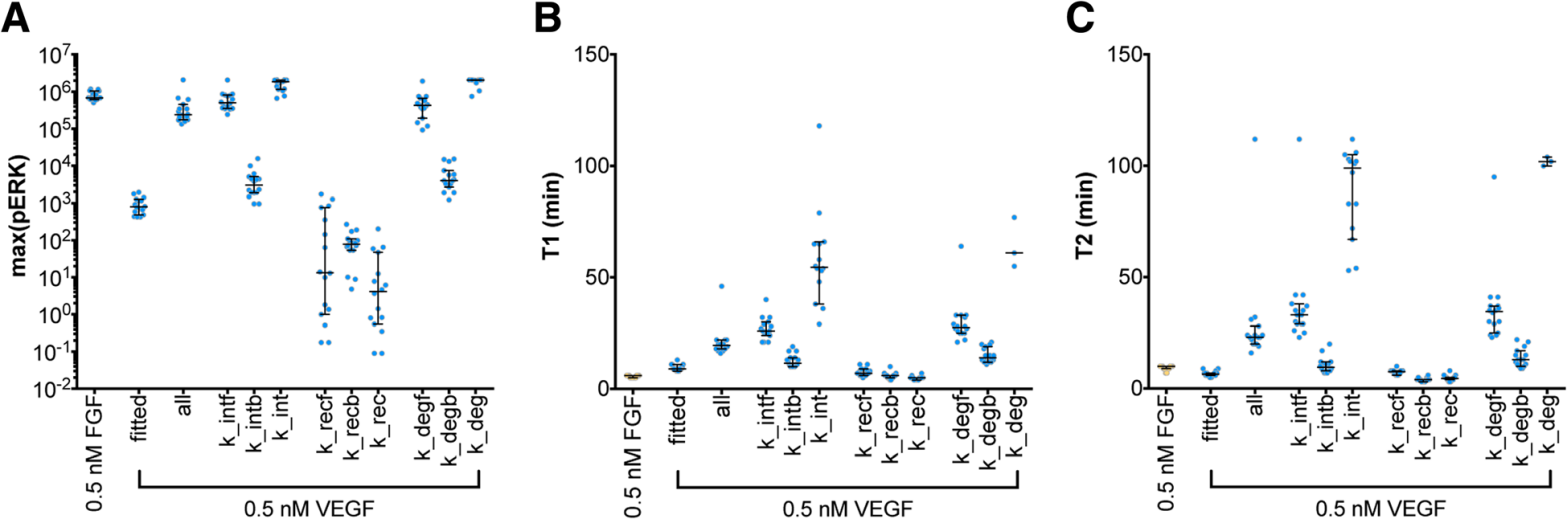
Effect of varying VEGFR2 trafficking parameters on pERK response. **a** Maximum pERK, **b** *T1*, and (**c**) *T2*. The panels show the effect of 0.5 nM FGF (Yellow) or 0.5 nM VEGF (Blue) predicted using the fitted parameter values (“*fitted*” *x*-axis label). We ran the model with 0.5 nM VEGF when all of the VEGFR2 trafficking parameters are decreased (“all” *x*-axis label) to be the same as the FGFR trafficking rates shown in Additional file 1: Figure S5. Finally, we decreased each VEGFR2 trafficking parameter individually to be the same as the corresponding FGFR trafficking rate shown in Additional file 1: Figure S5. We omitted some points for T1 and T2 when the pERK does not reach the maximum value in two hours. Each dot represents one fit. Bars are median ± 95% confidence interval

Because FGFR trafficking parameter values are lower than the corresponding VEGFR2 trafficking parameters (Additional file 1: Figure S5) and pERK is sensitive to these trafficking rates (Additional file 1: Figure S1), we explored the role of trafficking parameters in pERK response. That is, we investigated the effects of decreasing the VEGFR2 trafficking parameters individually or together to the same level as FGFR trafficking parameters (Fig. 7). Specifically, we simulated the signaling dynamics with 0.5 nM VEGF when all of the VEGFR2 trafficking parameters are decreased to be the same as the FGFR trafficking rates shown in Additional file 1: Figure S5. We then decreased each VEGFR2 trafficking parameter one-by-one to be the same as the corresponding FGFR trafficking rate shown in Additional file 1: Figure S5. By performing these simulations, we can determine how the trafficking of each pool of VEGFR2 molecules influences the response to VEGF stimulation.

We found that decreasing the internalization rates of the free and bound forms of VEGFR2, individually or in combination, with 0.5 nM VEGF leads to a significant increase in maximum ERK phosphorylation (Fig. 7a, “*fitted*” compared to “*k_intf*”, “*k_intb*”, or “*k_int*”). In fact, by decreasing VEGFR2 internalization (“*k_int*”) to be the same as the FGFR internalization rate, the maximal VEGF-mediated ERK phosphorylation reaches the same magnitude as the response induced by 0.5 nM FGF. This qualitative trend is expected, since decreasing receptor internalization rates makes more VEGFR2 available on the cell surface to bind to the ligand, inducing downstream signaling. However, the model provides detail about the quantitative effects of these changes.

Decreasing the recycling rates of the free and bound forms of VEGFR2 individually or in combination significantly decreases ERK phosphorylation for 0.5 nM VEGF stimulation (Fig. 7a, “*fitted*” compared to “*k_recf*”, “*k_recb*”, or “*k_rec*”). A lower receptor recycling rate makes more non-signaling internalized VEGFR2 remain inside the cell longer and recycles available VEGFR2 to the cell surface more slowly. Together, these effects limit ERK phosphorylation. Interestingly, changing the recycling rate leads to a wider range of responses compared to changing internalization or degradation rates, especially for recycling of free VEGFR2 (Fig. 7a).

Lowering the rate at which the free form of VEGFR2 is degraded significantly increases the maximum ERK phosphorylation induced by 0.5 nM VEGF to be the same magnitude of the maximal pERK level induced by 0.5 nM FGF alone (Fig. 7a, “*fitted*” compared to “*k_degf*”, “*k_degb*”, or “*k_deg*”). A lower free receptor degradation rate makes more VEGFR2 available to promote signaling.

We also examined the timescales of the ERK response. The model predicts that decreasing the trafficking rates slows down the dynamics of VEGF-induced ERK phosphorylation (Fig. 7b, c), both in terms of T1 and T2. Furthermore, we studied the effects of changing VEGFR2 trafficking parameters on the combination effects. We found that the combination of 0.5 nM FGF and 0.5 nM VEGF with lower VEGFR2 trafficking parameters has similar results as 0.5 nM VEGF-induced pERK. That is, decreasing VEGFR2 internalization and degradation rates leads to greater ERK phosphorylation (Additional file 1: Figure S11). Overall, lower VEGFR2 trafficking parameters leads to an increased impact of VEGF in the combination effects.

## Discussion

We developed an intracellular signaling model of the crosstalk between two pro-angiogenic factors, FGF and VEGF. The molecular-detailed model represents the reaction network of interactions on a molecular level, based on reactions documented in literature. The kinetic parameters are taken from experimental measurements, where available. Unknown parameters were estimated by fitting the model to experimental data. Additionally, we validated the model using three separate sets of data.

This is a novel model of FGF and VEGF interactions, taking into account previous modeling work [6, 16], with a focus on the MAPK cascade and the pERK response as an indicator for pro-angiogenic signaling. The fitted model predicts the pERK response upon stimulation by FGF and VEGF, alone and in combination. We particularly focus on the pERK response since pERK promotes cell proliferation [14], one aspect of early-stage of angiogenesis. Additionally, it has been shown that pERK is mostly found in active rather than quiescent endothelial cells [15], and pERK has been used as way to characterize the pro-angiogenic response in other studies [27–29].

Overall, FGF is predicted to potently and rapidly promote ERK phosphorylation compared to VEGF stimulation. VEGF also plays an important role in pERK dynamics. Altogether, the model shows that the pERK level in response to FGF, VEGF, and their combination is dose-dependent and that some combinations induce greater maximum ERK phosphorylation than the summation of their individual effects.

Our results reveal that the strength of VEGF-mediated ERK signaling is a combination of the absolute receptor expression level, the receptor availability, and some intrinsic characteristic of the receptors or the structure of the signaling pathway. Firstly, there is imbalanced receptor expression level (high FGFR1 compared to VEGFR2), which is one of the reasons for VEGF’s lower ERK activation in HUVECs. Increasing the expression of VEGFR2 by five-fold (from 1000 up to 5000 receptors/cell), without changing VEGFR2 trafficking parameters allows the maximum VEGF-induced pERK level to be the same as what is achieved through FGF stimulation at equimolar concentrations. Additionally, changing the VEGFR2 trafficking rates to be the same as those for FGFR, without changing VEGFR2 density, also allows the same maximal pERK to be achieved for equimolar concentrations of FGF and VEGF. Thus, both the absolute receptor level and availability of receptors directly affect the signal strength. However, these are not the only causes of the difference in the ability to drive ERK signaling, because even with a five-fold increase in VEGFR2 (to 5000 molecules/cell), its expression is still less than that of FGFR, which is present at 20,000 molecules/cell. Thus, to reach the same maximum pERK level at equimolar concentrations of FGF and VEGF stimulation, the required VEGFR2 level is much lower than FGFR level, independent of the high VEGFR2 trafficking rates. This indicates that there is also some intrinsic ability of VEGFR2 that enables ERK activation. This is particularly relevant, as endothelial VEGF receptor expression is upregulated in tumors [30], and can reach nearly 2 × 10^4^ molecules/cell in tumor-derived endothelial cells after six weeks of tumor growth [26]. The effect of VEGF-mediated pERK signaling may also be due to the structure of the signaling network and expression levels of the pathway intermediates (such as Raf, which is not present in the FGF signaling pathway). Overall, the ability of VEGF to promote ERK signaling is due to a combination of factors. Excitingly, our model is able to predict the contribution of each of these factors.

The model predictions are consistent with several experimental studies. Multiple experiments show that FGF induces the same level of angiogenic response at lower concentration in comparison to VEGF [31–33], and their combination induces greater angiogenic responses [8]. Additionally, the model predicts that decreasing VEGFR2 internalization and degradation rates can increase the impact of VEGF in combination effects. This result complements experiments showing that receptor trafficking plays a critical role in angiogenic signaling [34]. Overall, our molecular-detailed model helps synthesize these experimental data and observations related to VEGF- and FGF-stimulated signaling.

One application of our work is that the model can also be linked with computational models that predict events on the cellular scale. Our model culminates with ERK activation, complementing published models that substantially simplify the intracellular signaling and focus on specific cellular behavior, such as proliferation [35], the probability of sprout formation and the speed of vessel growth [36], or tumor growth [37]. However, these models reduced the intracellular signaling network such that the output signal is simply linearly proportional to the fraction of bound receptors. In comparison, our mechanistic model considers intracellular signaling and quantitatively analyzes pERK response, which could be a better indicator for these cellular behaviors. For example, Hendrata and Sudiono constructed a computational model that includes molecular, cellular, and extracellular scales to study tumor apoptosis [37]. Our model can be utilized in combination with such models to more accurately predict cellular behavior.

Our model can also be used for exploring mechanisms that regulate the magnitude and dynamics of pERK upon FGF and/or VEGF stimulation, as has been done in other modeling work [38–40]. For example, Edelstein et al. showed that ligand depletion diminishes cooperative interactions between ligands and binding sites, and that receptor concentration plays an important role in biological signal transduction [38]. Such depletion of the ligand that initiates the signaling could also be explored using our mechanistic model. In other work, Saucerman and Bers combined a cardiac myocyte excitation-contraction computational model with biochemical reaction models to investigate how calmodulin (CaM), calcineurin, and CaM-dependent kinase are spatially and temporally activated by local calcium signals [39]. Our model can be expanded to explore spatial effects as well. Recently, Romano and coworkers studied the competition of seven proteins for CaM binding and concluded that this competition contributes to synaptic plasticity [40]. This model of binding competition is relevant to our system, for example in the case of competitive binding the activators (FRS2 or aRaf) and phosphatases to species such as MEK. Such competition can be examined in detail in future work.

Our model can also aid in studying the efficiency of pro- or anti-angiogenic therapies. Some pro- or anti-angiogenic treatments have not been very effective, particularly those targeting only a single signaling family [2, 3]. However, targeting both FGF and VEGF may be a promising strategy, given the potential synergistic effects predicted by our model and demonstrated in experimental studies. In fact, multiple groups have reported interesting interactions between FGF and VEGF [7, 8, 41, 42]. This crosstalk may be exploited to aid in angiogenesis-based therapies, and our model can be helpful in understanding their interactions and combination effects. Model predictions for species’ dynamics and reaction rates provide mechanistic insight into FGF and VEGF interactions. Our predictions show that the low success in targeting VEGF alone could be due to low receptor numbers and fast internalization, recycling and degradation. Although FGF has greater effects in inducing ERK phosphorylation, its effects can be enhanced by the addition of VEGF. Thus, our model can be used to investigate the efficiency of targeting both FGF and VEGF as an alternative strategy.

In addition to the amount of FGF or VEGF the cells are stimulated with, other factors can influence the magnitude of timescale of the pERK response. This includes the growth factor concentration gradient, heterogeneity in a population of cells, and genetic mutations. Our computational modeling provides a platform for many interesting and relevant studies that can be helpful to characterize signaling dynamics that mediate endothelial cell sprouting during the early stages of angiogenesis, in response to extracellular signals.

Our model is the first to combine the signaling networks of FGF and VEGF, providing novel quantitative insight into the effect of combined FGF and VEGF treatment. However, we recognize some limitations in our model. Firstly, this model does not include FGF activated Ras-Raf signaling because the protein-protein interactions in this pathway are still not clear. As more information becomes available as to the detailed mechanisms of those reactions, we can expand the model to include FGF-mediated Ras signaling. Second, we assumed that the internalized and degraded phosphorylated receptors would not signal. Receptor trafficking processes such as internalization have conventionally been thought to downregulate extracellular signals. However, data suggest that VEGFR2 may signal even when internalized [43–45]. Since the role of internalized receptors is still somewhat debated and to focus on the signaling dynamics mediated by cell surface receptors, we chose to exclude the effect of internalized receptors in our model. This assumption can be relaxed in future studies. Additionally, all bound forms of FGFR1 are assumed to have the same internalization, recycling, and degradation rates as a simplification and because there are conflicting values reported in literature [20, 46]. We tried various FGFR1 trafficking parameters; however, this did not significantly change the model predictions or our overall conclusions. Third, this model only includes VEGFR2, although VEGF binds to VEGFR1 and neuropilin-1 (NRP1). These receptors also contribute to angiogenesis and may be incorporated into the model in future studies. Finally, we studied pERK response in two hours. We omit ligand secretion and protein degradation during this time and do not predict long-term responses. In the future, we can expand our model to predict the cellular response over a longer period of time.

## Conclusions

In summary, our molecular-detailed model quantifies ERK phosphorylation upon stimulation by two major pro-angiogenic factors, FGF and VEGF, and provides insights into the molecular interactions between these proteins. Specifically, the model predicts the combination effects of FGF and VEGF on ERK phosphorylation and quantitatively shows the magnitude and time scale of the pERK response. Because of the complexity of this biological system, it may be challenging to get a comprehensive understanding of the system using experiments that only focus on a few molecular species. Our computational modeling provides a quantitative framework to explore the system as a whole, generating novel mechanistic insight and complementing experimental studies.

## Methods

### Model construction

We constructed a molecular-detailed biochemical reaction network including FGF, VEGF, and their receptors FGFR1 and VEGFR2 (Fig. 1). Signaling is induced by the growth factors binding to their receptors, culminating with phosphorylation of ERK, through the MAPK cascade. MAPK signaling is initiated through the activation of Raf and FRS2 by VEGF and FGF, respectively. Activated Raf (aRaf) and phosphorylated FRS2 (pFRS2) phosphorylate MEK at two sites, and doubly phosphorylated MEK (ppMEK) further phosphorylates ERK. The molecular interactions involved in the network are illustrated in Fig. 1. The model is a novel advancement of published computational models [6, 16]. Specifically, we adapted the competition of FGF and HSGAG to the binding of FGFR1 and the feedback loop from pERK to FRS2 from the model by Kanodia et al., and we expanded the model by including FGFR trafficking (internalization, recycling, and degradation) and accounting for both singly- and doubly-phosphorylated MEK and ERK. It is worth noting that the formation of the tertiary signaling complex of FGF:HSGAG:FGFR only occurs by FGFR binding to the FGF:HSGAG complex. This is because the affinity of FGF:HSGAG binding is approximately two times stronger than that of FGF:FGFR, and FGFR and HSGAG binding is more than two orders of magnitude lower [47]. In addition, we simplified the model of VEGF-induced ERK phosphorylation pathways from Tan and coworkers; specifically, we only include Ras activation either from Shc-independent or Shc-dependent pathways [6]. Thus, we expanded upon previous models to capture the major steps of FGF- and VEGF-induced ERK phosphorylation and better understand their interactions.

The model is simulated using a range of concentrations for FGF and VEGF, based on published experimental studies. Typically, FGF and VEGF concentrations are within the range of 0 to 50 ng/ml (0–2.2 nM) [31, 33, 48] and 0 to 100 ng/ml (0–2.2 nM) [17, 18, 25, 49, 50], respectively, in in vitro studies, although some studies utilized concentrations as high as 300 ng/ml VEGF [8] and 500 ng/ml FGF [16]. It has been reported that 25 ng/ml (0.56 nM) and 50 ng/ml (1.1 nM) VEGF significantly increase tube formation by HUVECs, and 0.1 ng/ml (0.004 nM) and 1.0 ng/mL (0.04 nM) FGF strongly induced tube formation on Matrigel after 24 h compared to the control groups [33]. Moreover, Pepper et al. showed that the total sprout length formed by bovine microvascular endothelial cells started to plateau when treated with 30 ng/ml (1.3 nM) FGF and 100 ng/ml (2.2 nM) VEGF [8]. To account for these findings, we simulated the model with the concentration of FGF and VEGF ranging of 0.01 to 2 nM.

The network is implemented as an ordinary differential equation (ODE) model using MATLAB (Mathworks, Inc.). The main model includes 70 reactions, 72 species, and 75 parameters. The reactions, initial conditions, and parameter values are listed in Additional file 2: Tables S2 to S4. All reactions are assumed to follow the law of mass action. Receptor internalization, recycling, and degradation are considered in the model. Because the simulated time is within two hours, we do not consider the degradation of the ligands or signaling species. The final model is available in Additional file 3. We also implement a modified model that includes heparin to validate the estimated model parameters (described below).

### Sensitivity analysis

To identify the parameters and initial concentrations that significantly influence the model outputs, we performed the extended Fourier Amplitude Sensitivity Test (eFAST) [51]. All targeted parameters and initial values were varied simultaneously within specified bounds (one order of magnitude above and below the baseline values), and the effects of multiple model inputs (kinetic parameters or initial conditions) on the pERK concentration were computed (the total sensitivity indices, “*S*_ti_”). We studied the *S*_ti_ values for kinetic parameters and initial concentrations. The *S*_ti_ index can range from 0 to 1, where a higher *S*_ti_ index indicates this input is more influential to the output.

### Data extraction

Data from published experimental studies [16–18, 24, 25] were used for parameter fitting and model validation. The Western blot data was extracted using ImageJ. Experimental data from plots was extracted using the function *grabit*.

### Model parameters

The trafficking parameters for VEGFR2 and the parameters and initial values that are involved in the overlap of FGF and VEGF signaling pathways were estimated by fitting the model to experimental data using Particle Swarm Optimization (PSO) implemented by Iadevaia [19]. We used MATLAB to implement the PSO algorithm. A total of 39 parameters and initial values were estimated in the fitting (Additional file 2: Table S1, and also highlighted in red in Additional file 2: Tables S3 and S4). All other parameters were taken from published literature [6, 16, 20]. The parameters characterizing the overlapping MAPK pathway were chosen for fitting because while FGF and VEGF upstream parameters are well documented individually in literature, a uniform set of parameters for their interactions is needed for this combined model. The VEGFR2 trafficking parameters were fitted, as they have been shown to significantly affect ERK activation [6]. Additionally, many of the kinetic parameters for the overlapping reactions and the VEGFR2 trafficking rates were shown to significantly influence pERK in the sensitivity analysis (Additional file 1: Figure S1).

PSO starts with a population of initial particles (parameter sets). As the particles move around (i.e., as the algorithm explores the parameter space), an objective function is evaluated at each particle location. Particles communicate with one another to determine which has the lowest objective function value. The objective function for each parameter set was used to identify optimal parameter values. Specifically, we used PSO to minimize the weighted sum of squared residuals (WSSR):

$$ \mathrm{WSSR}\left(\uptheta \right)=\mathit{\min}\sum \limits_{i=1}^n{\left(\frac{V_{pred,i}\left(\uptheta \right)-{V}_{\mathit{\exp},i}}{V_{\mathit{\exp},i}}\right)}^2 $$

where V_*exp,i*_ is the *i*th experimental measurement, V_*pred,i*_ is the *i*th predicted value at the corresponding time point, and *n* is the total number of experimental data points. The minimization is subject to *θ*, the set of upper and lower bounds on each of the free parameters. The bounds were set to be one order of magnitude above and below the baseline parameter values, which were taken from literature and listed in Additional file 2: Tables S3 to S4. Although PSO is a global parameter estimation approach, and the parameter values are varied within each run to minimize the error, we still ran the algorithm multiple times to attempt to identify the optimal parameter values within the large search space. We were able to obtain a total of 72 fitted parameter sets, which were ultimately narrowed down to 16 parameter sets that allowed the model to capture the training and validation data sets.

The model was fitted against three datasets, specifically: 1) normalized pERK induced by FGF concentrations varying from 0.16 ng/ml to 500 ng/ml, where pERK level was normalized by the maximum pERK stimulated by FGF across all six concentrations (0.16, 0.8, 4, 20, 100, and 500 ng/ml) in two hours, experiments conducted using the H1703 cell line [16]; 2) normalized pVEGFR2 (pR2) stimulated by 5 ng/ml VEGF, pR2 was normalized by the maximum pR2 induced by 5 ng/ml VEGF, experiments conducted using HUVECs [18]; 3) normalized pERK induced by 50 ng/ml VEGF, where pERK was normalized by the maximum pERK induced by 50 ng/ml VEGF, experiments conducted using HUVECs [17]. We note that the pERK and pR2 in the model simulation include all free and bound forms of pERK and ppERK, and all free and bound forms of pR2 except the degraded pR2, respectively.

After model training, we validated the fitted model with three datasets not used in the fitting. First, we simulated the effects following the addition of heparin. For this case, we added 500 μg/ml heparin, which competes with HSGAG and binds to FGF. There are an additional 26 reactions, 25 species, and 3 parameters for heparin perturbation in the model. Without any fitting, parameters are all taken from Kanodia et al.. The influence of heparin is illustrated in Additional file 1: Figure S3, and details are provided in Additional file 2: Tables S5 and S6. We simulated the pERK dose response with or without heparin to compare with the experiments described by Kanodia [16]. Having a difference in pERK with and without additional heparin that is greater than zero indicates that the presence of heparin enhances FGF-induced ERK phosphorylation. Second, we predicted the phosphorylated pERK following stimulation with 10 ng/ml FGF, mimicking measurements obtained from BAECs [24]. Third, we predicted the VEGF-induced pR2 response upon stimulation with 80 ng/ml VEGF, simulating experiments conducted using HUVECs [25]. For all three datasets, we simulated the experimental conditions without any additional model fitting and compared to the experimental measurements. A total of 16 parameter sets with the smallest errors were taken to be the “best” sets based on the model fitting and validation (Additional file 1: Figure S2 and Additional file 2: Table S1) and were used for model simulations.

#### VEGFR2 density

To study the impact of VEGFR2 expression on VEGF-induced angiogenesis, we varied VEGFR2 density within ten-fold of the baseline value (1000 molecules/cell) and predicted the level and dynamics of ERK phosphorylation.

#### VEGFR2 trafficking parameters

To investigate the effects of VEGFR2 trafficking in VEGF-induced ERK phosphorylation, we decreased the trafficking parameters (internalization, recycling, and degradation rates) values for VEGFR2. We changed the parameters one-by-one or together to be the same level as FGFR trafficking parameters and predicted the VEGF-induced pERK response. For example, we made the internalization rate of free VEGFR2 to be the same as the rate at which free FGFR is internalized.

### ERK phosphorylation response

We investigated the ERK phosphorylation response by the stimulation of FGF or VEGF alone, compared to their combination. In this study, we mainly focus on two aspects of pERK dynamics: magnitude of the response and timescale of signaling.

#### Magnitude of ERK phosphorylation response

- *Maximum pERK*. We calculate the maximum ERK phosphorylation level induced by the stimulation of FGF, VEGF, or their combination.
- *Ratio, R*. To compare the combination effects with FGF and VEGF individual effects, we introduce the ratio below:

$$ R=\frac{\mathit{\max}\ \mathrm{pERK}\left(\mathrm{FGF}\ \mathrm{and}\ \mathrm{VEGF}\right)}{\mathit{\max}\ \mathrm{pERK}\left(\mathrm{FGF}\right)+\mathit{\max}\ \mathrm{pERK}\left(\mathrm{VEGF}\right)} $$

When *R* is greater than one, it indicates that the combination effect in inducing maximal pERK is greater than the summation of individual effects; when *R* is equal to one, it implies that the combination effect is additive; when *R* is less to one, it suggests an antagonistic effect between FGF and VEGF.

#### Timescale of the signaling response

We use two parameters to characterize the timescale of ERK activation: the time to reach the maximum pERK (*T1*) and the time duration that pERK level remains greater than half of its maximal value (*T2*). T1 indicates how quickly ERK is phosphorylated: the smaller T1 is, the faster ERK becomes phosphorylated. T2 indicates how long ERK remains in a phosphorylated state: the larger T2 is, the more sustained the pERK response.

#### Reaction rates

We specify the rates of each reaction based on the law of mass action, where the rate of a chemical reaction is proportional to the amount of each reactant. For example, for the phosphorylation of VEGFR2:

$$ VEGF+ VEGFR2\ \overset{k_{pR2},\kern0.75em {k}_{dpR2}}{\leftrightarrow }\  pVEGFR2 $$

The reaction rate is:

$$ Rate={k}_{pR2}\bullet \left[ VEGF\right]\bullet \left[ VEGF R2\right]-{k}_{dpR2}\bullet \left[ pVEGFR2\right] $$

where *k*_*pR*2_ and *k*_*dpR*2_ are rate constants for the forward and reverse reactions, respectively, and [VEGF], [VEGFR2], and [pVEGFR2] are the species’ concentrations.

We simplified the VEGFR phosphorylation into one step because it has been reported that the two VEGFR2 monomers phosphorylate each other upon ligation [30]. Also, the specific mechanism of VEGFR2 phosphorylation is not our focus in this study. Therefore, we assume autophosphorylation upon VEGF binding, as implemented in other papers [6, 52].

## Additional files

Additional file 1: Supplementary figures. (PDF 2453 kb)

Additional file 2: Supplementary tables. (XLSX 32 kb)

Additional file 3: MATLAB file containing computational model. (DOCX 15 kb)
