# Supplementary material for: Mechanistic insight into activation of MAPK signaling by pro-angiogenic factors
Source: BMC Syst Biol. 2018 Dec 27;12:145. doi: 10.1186/s12918-018-0668-5 (PMC6307205; doi:10.1186/s12918-018-0668-5)
Supplement: Supplementary file 1 — Supplementary figures. (PDF 2453 kb) [file 12918_2018_668_MOESM1_ESM.pdf]

Figure S1

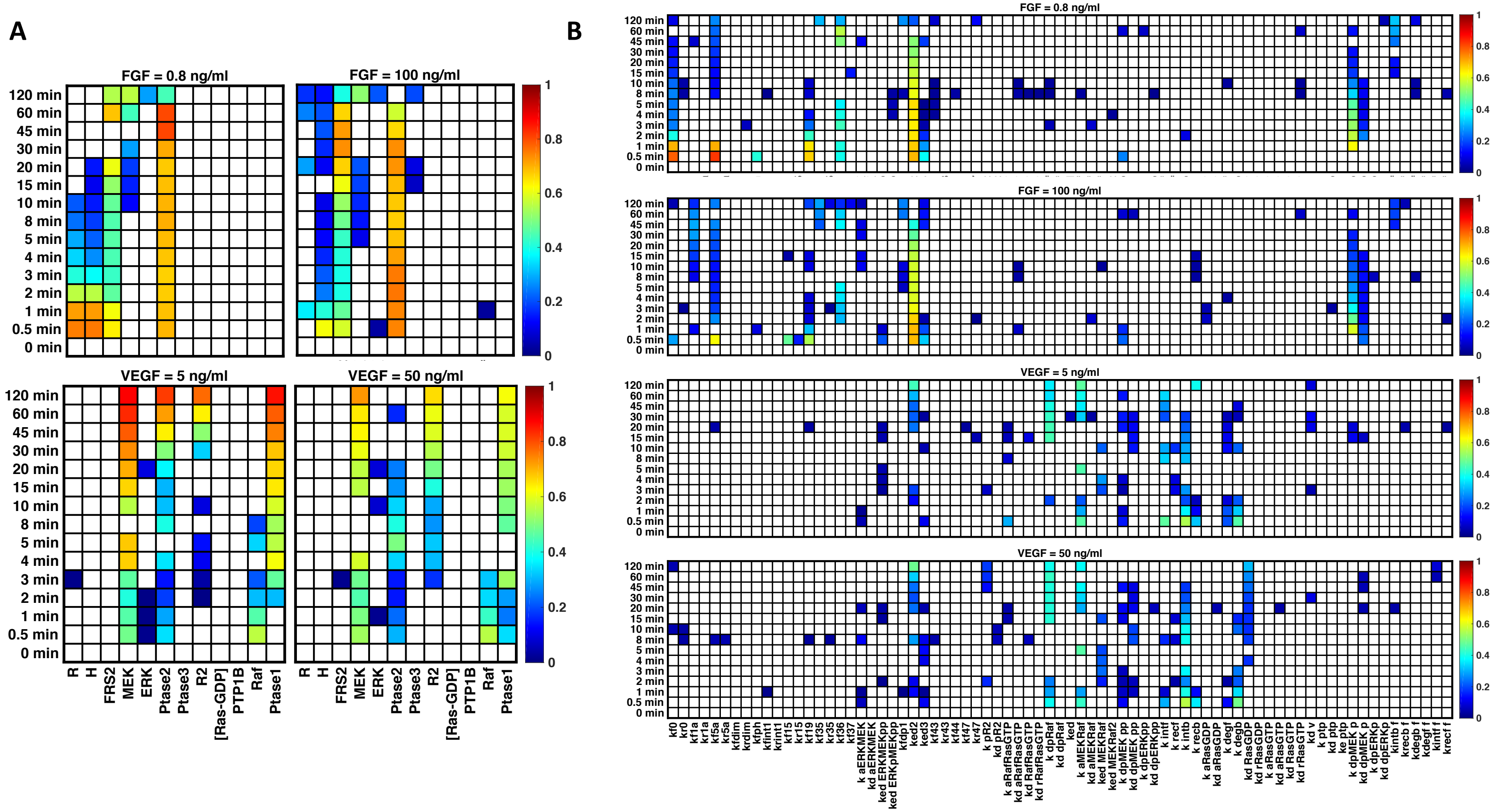

Figure S1. Results of eFAST sensitivity analysis. Total sensitivity index (S_{ti}) values for (A) 12 initial concentrations and (B) 75 parameters quantify how these inputs influence pERK level upon stimulation of FGF and VEGF. The color bars indicate the S_{ti} values.

Figure S2

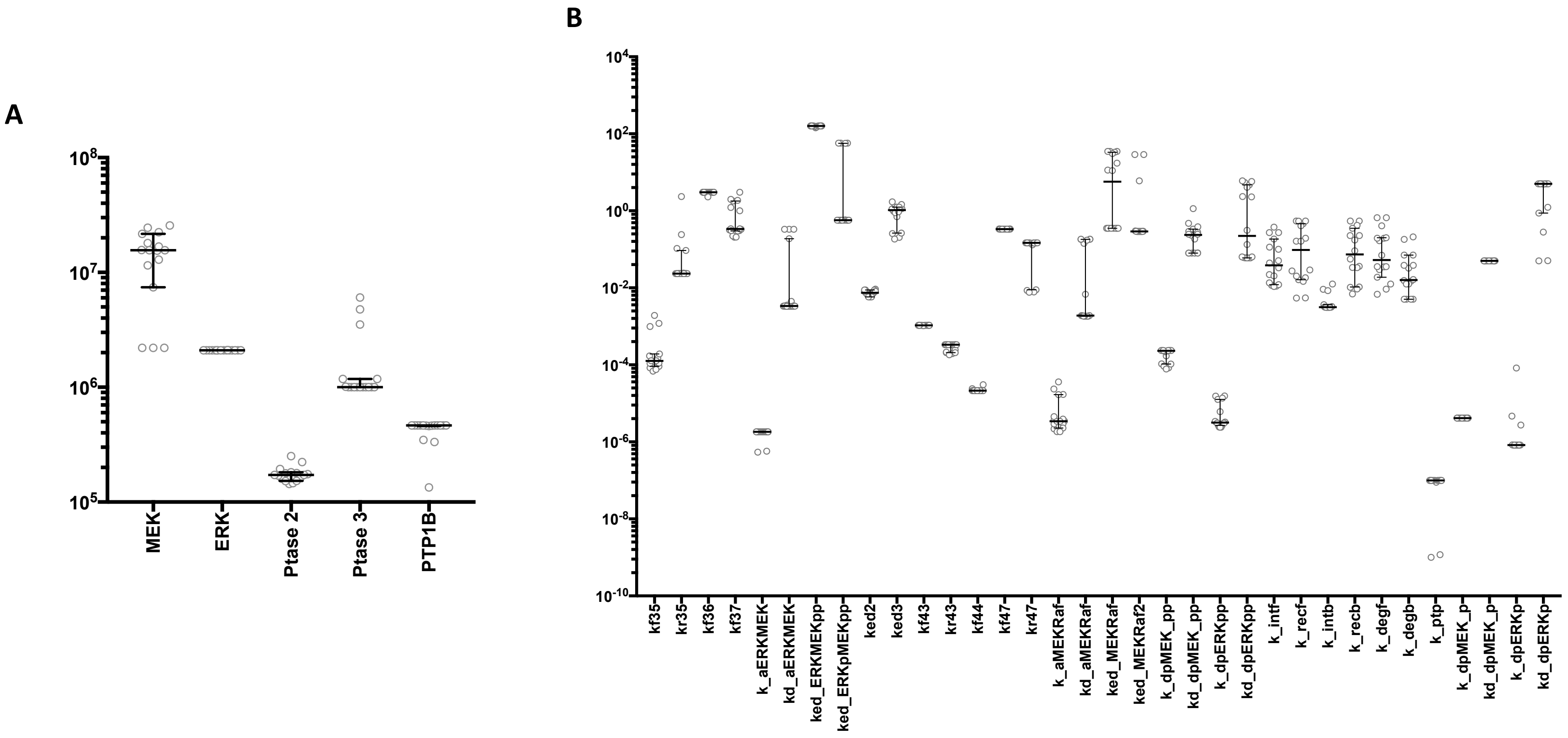

Figure S2. Model values estimated in fitting. (A) Distribution of fitted initial concentrations. (B) Model parameters. Each circle represents one fit. Bars are median ± 95% confidence interval.

Figure S3

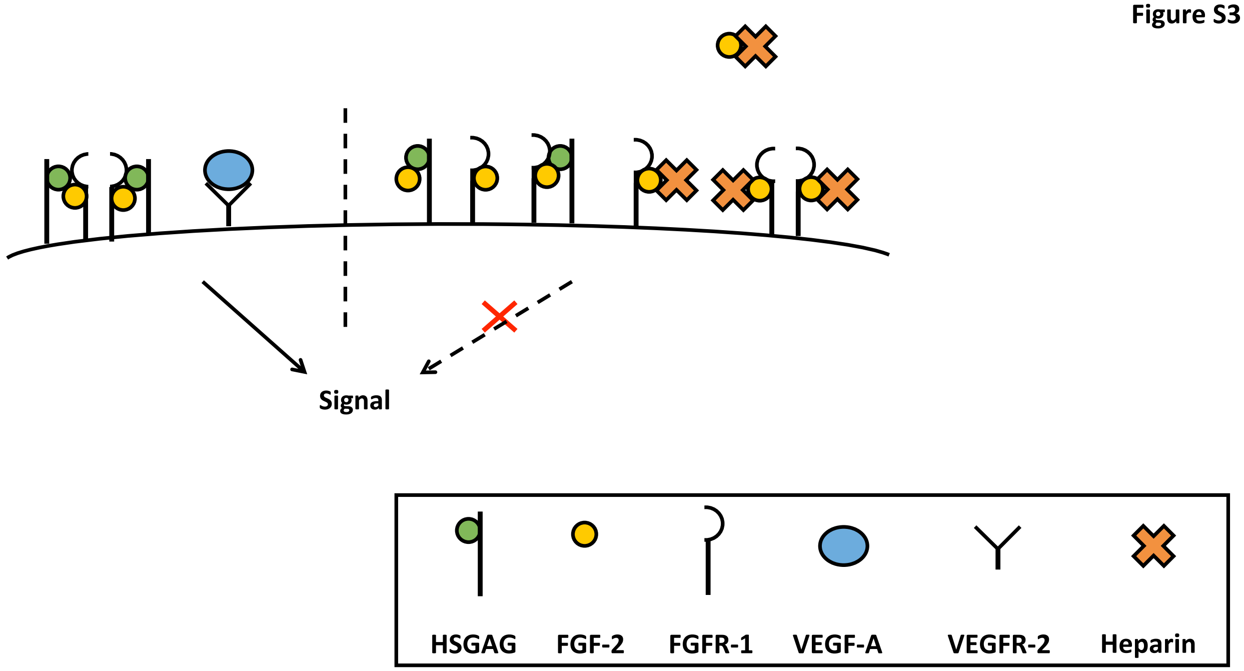

Figure S3. Schematic of ligand-bound receptors. Ligated FGFR1 and VEGFR2 promote downstream signaling. FGF:HSGAG:FGFR1 must dimerize. Complexes that include heparin do not induce signaling.

Figure S4

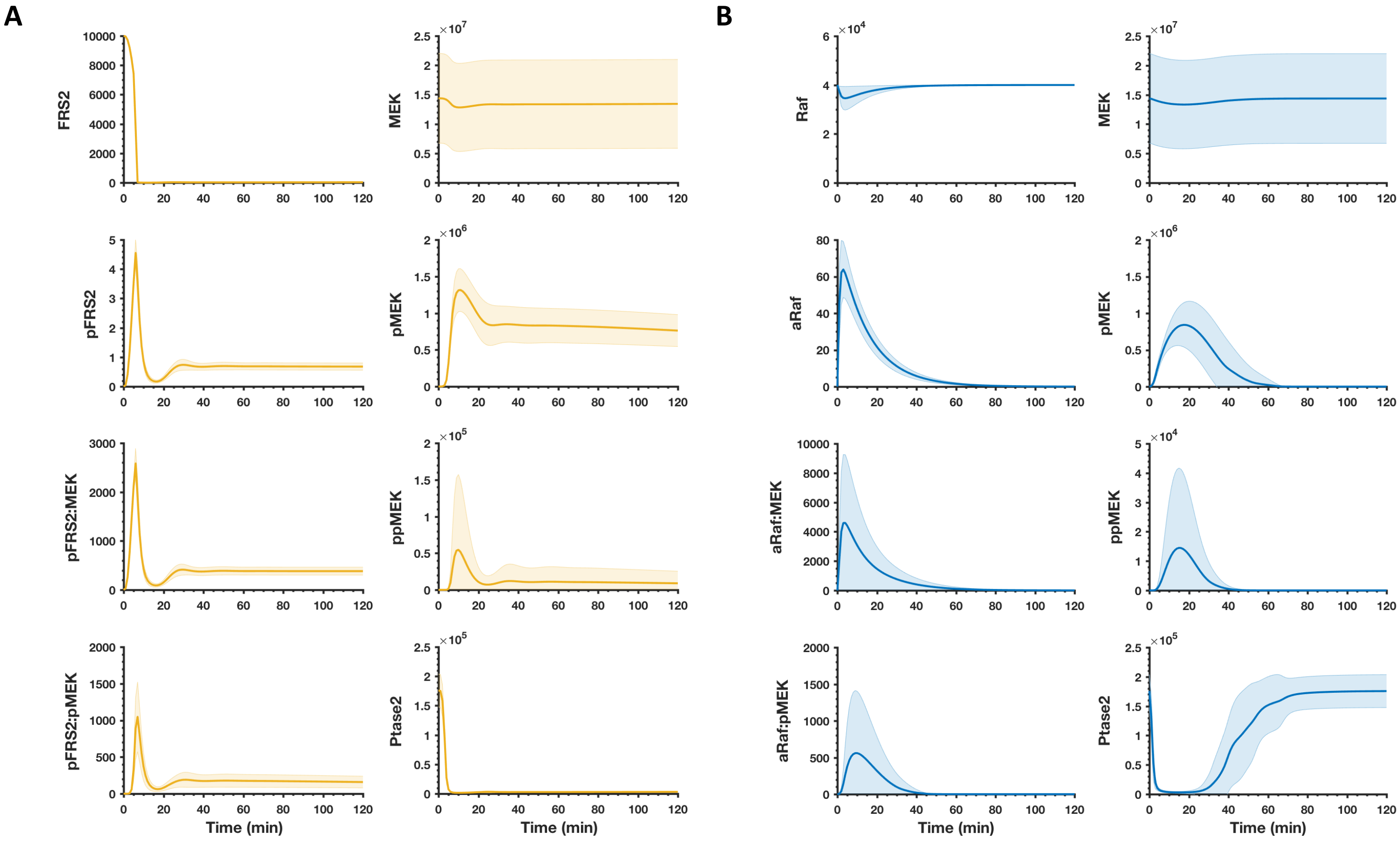

Figure S4. Dynamics of relevant species following stimulation with (A) 0.01 nM FGF or (B) 2 nM VEGF. Curves are the mean values of the 16 best fits, and shaded regions show standard deviation of the fits.

Figure S5

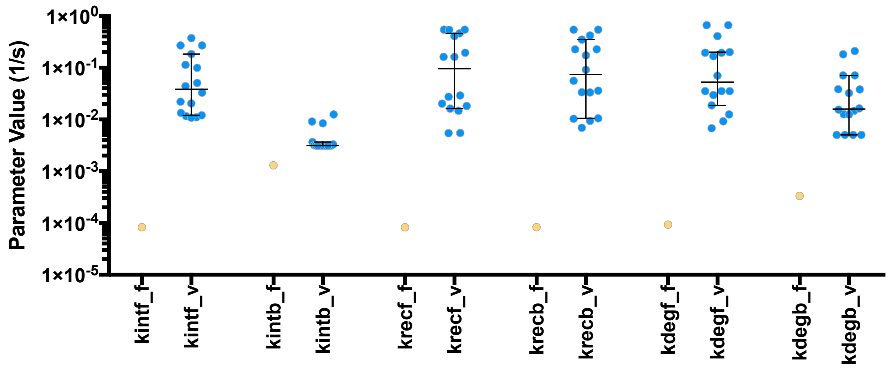

Figure S5. Distribution of estimated FGFR and VEGFR2 trafficking parameters. Yellow: FGF; Blue: VEGF (each dot represents one fit; bars are median ± 95% confidence interval).

Figure S6

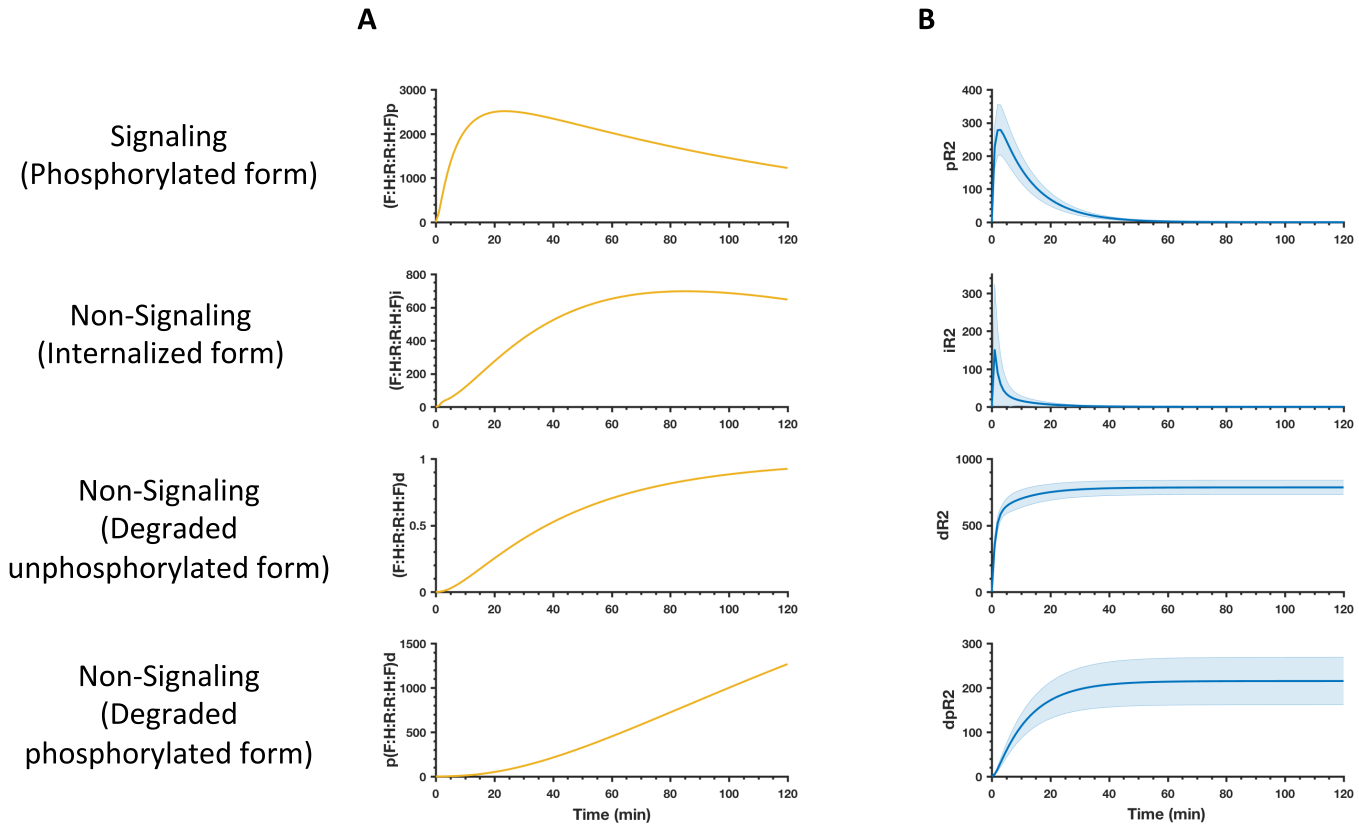

Figure S6. Predicted dynamics of relevant species following stimulation with (A) 0.5 nM FGF or (B) 0.5 nM VEGF. Curves are the mean values of the 16 best fits, and shaded regions show standard deviation of the fits.

Figure S7

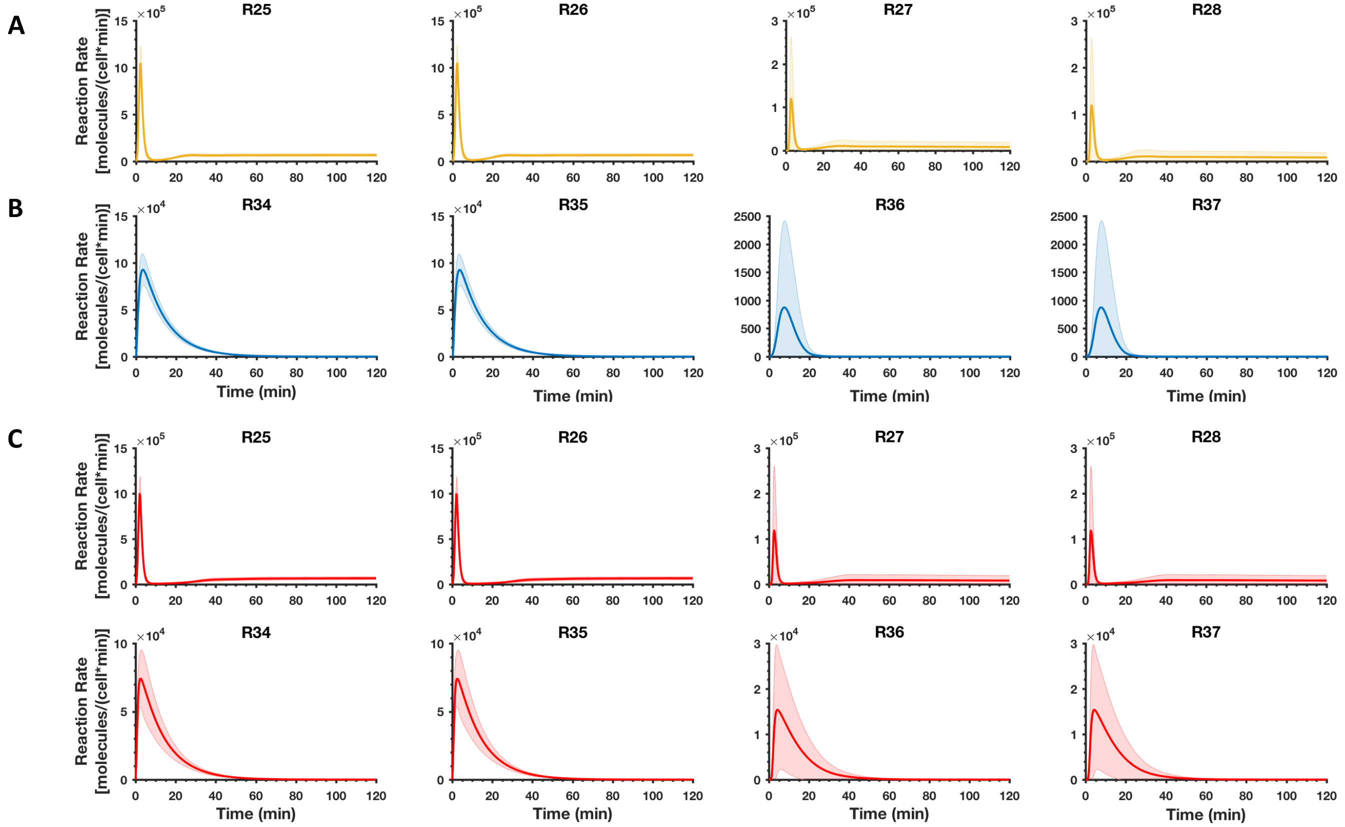

Figure S7. Reaction rates for MEK and pMEK phosphorylation following stimulation with (A) 0.5 nM FGF, (B) 0.5 nM VEGF, or (C) their combination. R25: FRS2p + MEK ↔ FRS2p:MEK; R26: FRS2p:MEK→FRS2p+pMEK; R27: FRS2p + pMEK ↔ FRS2p:pMEK; R28: FRS2p:pMEK → FRS2p + MEKpp; R34: MEK + aRaf ↔ MEK:aRaf; R35: MEK:aRaf → pMEK + aRaf; R36: pMEK + aRaf ↔ pMEK:aRaf; R37: pMEK:aRaf → ppMEK + aRaf. Yellow: FGF; Blue: VEGF; Red: combination. Curves are the mean values of the 16 best fits, and shaded regions show standard deviation of the fits.

Figure S8

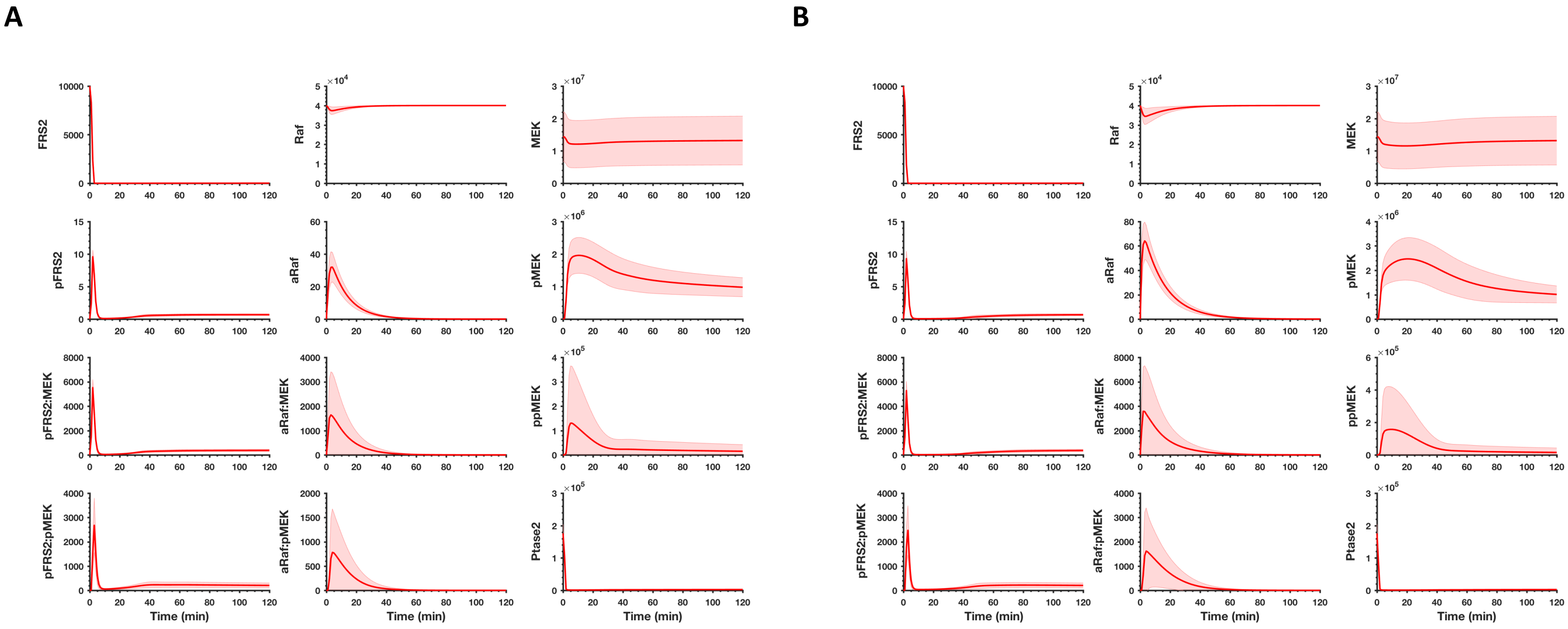

Figure S8. Dynamics of relevant species following stimulation with (A) 0.5 nM FGF and 0.5 nM VEGF in combination or (B) 0.5 nM FGF and 2 nM VEGF in combination (B). Curves are the mean values of the 16 best fits, and shaded regions show standard deviation of the fits.

Figure S9

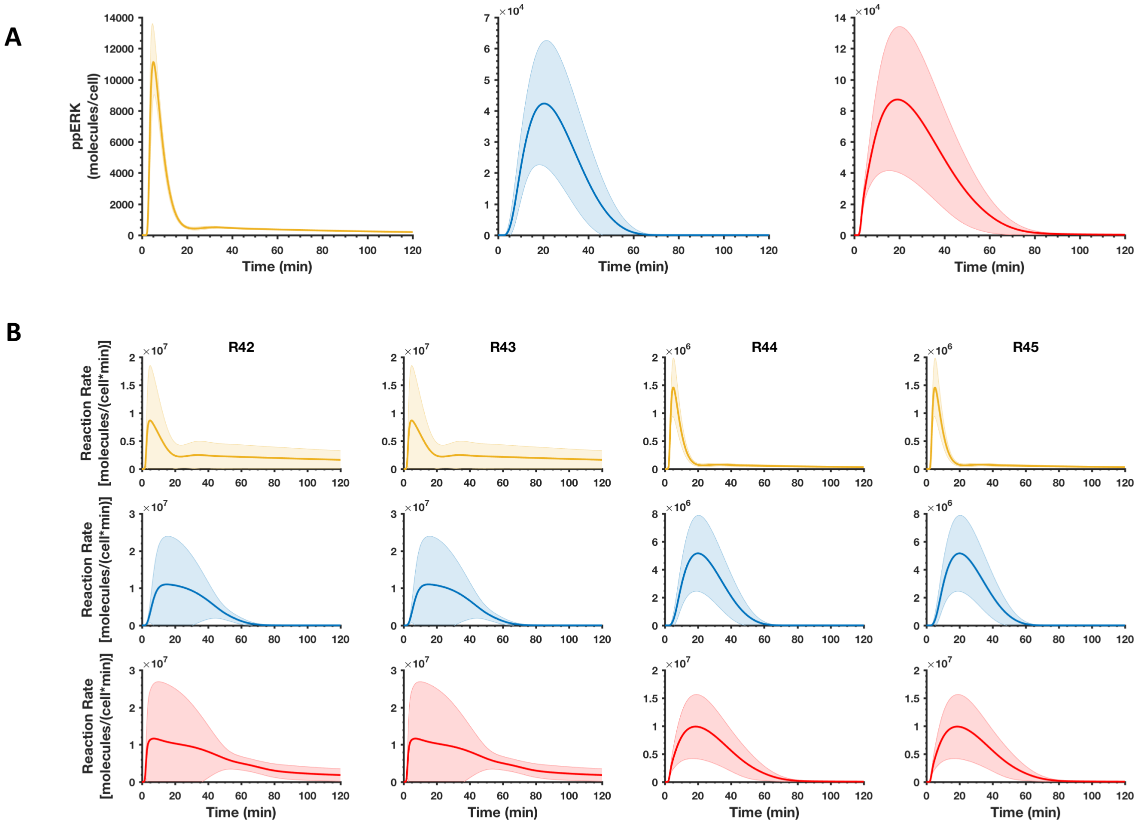

Figure S9. Reaction details in response to 0.5 nM FGF, 0.5 nM VEGF, and their combination stimulation with five-fold increase of VEGFR2 density. (A) Time course for ppERK dynamics. (B) Reaction rates for ERK and pERK phosphorylation. R42: ERK + ppMEK ↔ ERK:ppMEK; R43: ERK:ppMEK → pERK + ppMEK; R44: pERK + ppMEK ↔ pERK:ppMEK; R45: pERK:ppMEK → ppERK+ppMEK. Yellow: FGF; Blue: VEGF; Red: combination. Curves are the mean values of the 16 best fits and shaded regions show standard deviation of the fits.

Figure S10

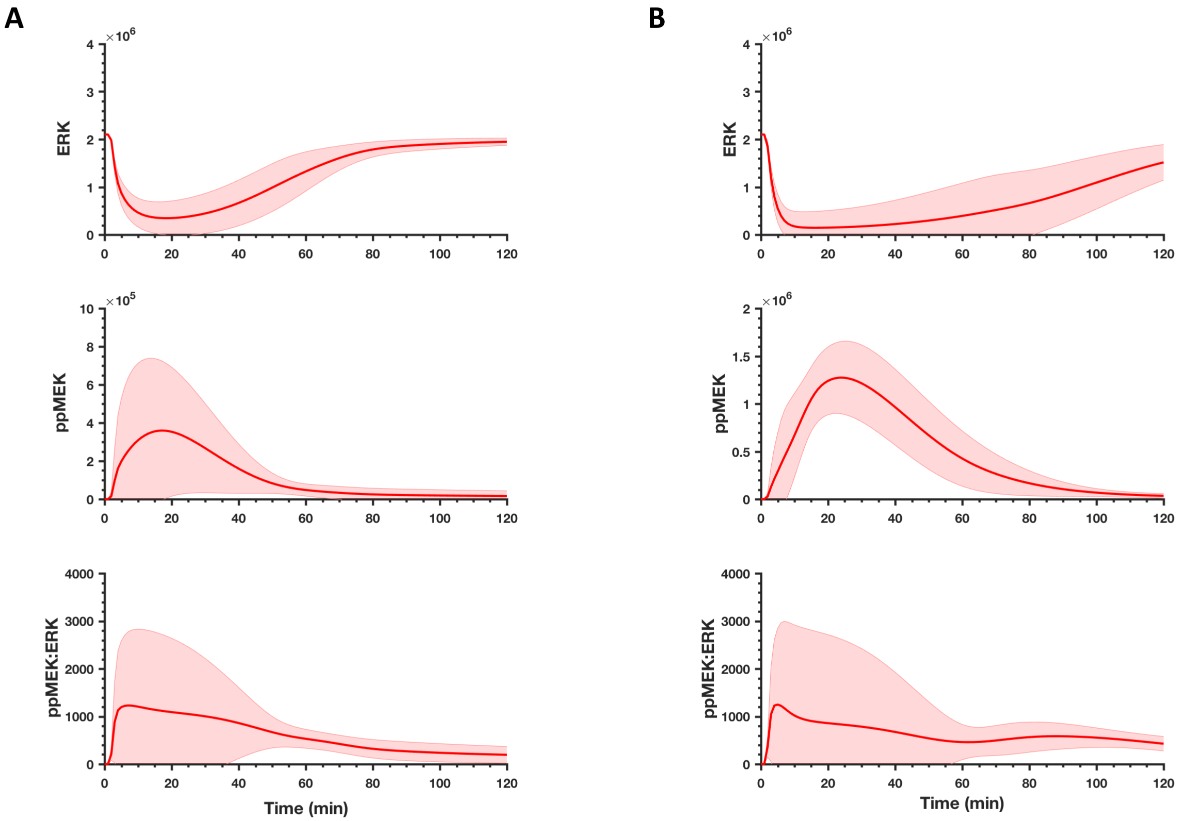

Figure S10. Dynamics of relevant species following stimulation by 0.5 nM FGF and 0.5 nM VEGF in combination with VEGFR2 density increased by (A) five-fold or (B) ten-fold. Curves are the mean values of 16 fits and shaded regions show standard deviation.

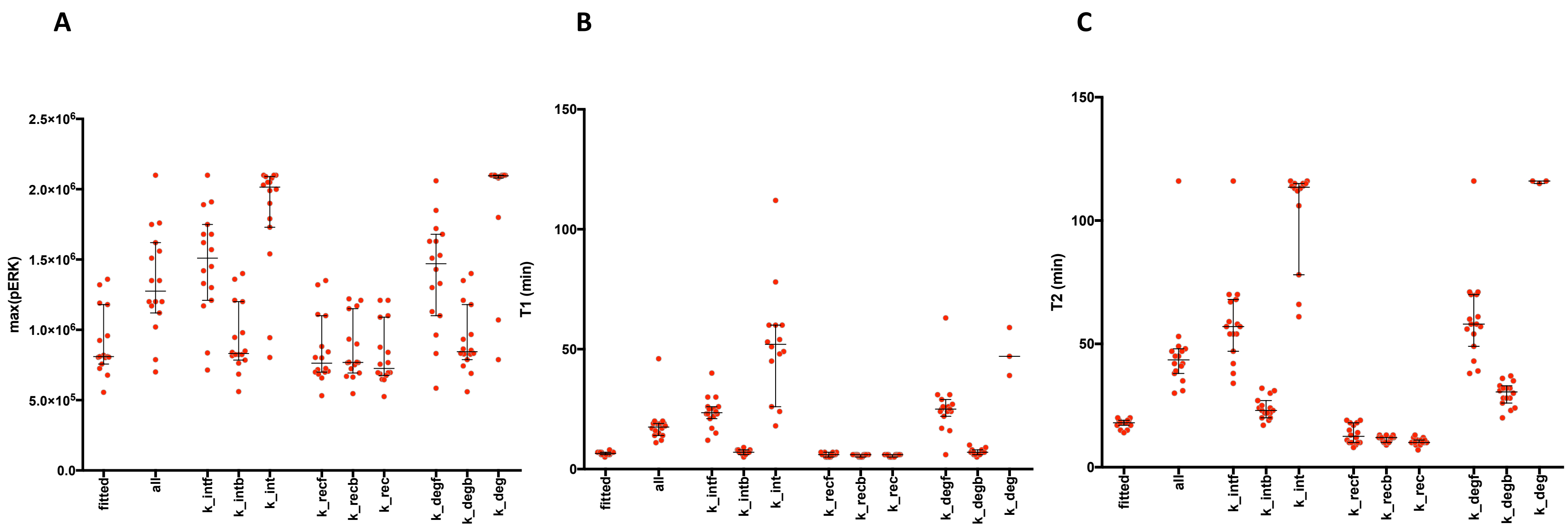

Figure S11. Effect of varying VEGFR trafficking parameters on pERK response. (A) Maximum pERK, (B) *T1*, and (C) *T2.* The panels show the effect of the combination of 0.5 nM FGF and 0.5 nM VEGF using the fitted parameter values ("fitted" *x*-axis label). We ran the model with FGF and VEGF combination when all of the VEGFR2 trafficking parameters are decreased ("all" *x*-axis label) to be the same as the FGFR trafficking rates shown in Figure S5. Finally, we decreased each VEGFR2 trafficking parameter individually to be the same as the corresponding FGFR trafficking rate shown in Figure S5. We omitted points for T1 and T2 when the pERK does not reach the maximum value in two hours. Each dot represents one fit. Bars are median ± 95% confidence interval.
