# Supplementary material for: Mechanistic insight into activation of MAPK signaling by pro-angiogenic factors
Source: BMC Syst Biol. 2018 Dec 27;12:145. doi: 10.1186/s12918-018-0668-5 (PMC6307205; doi:10.1186/s12918-018-0668-5)
Supplement: Supplementary file 3 — MATLAB file containing computational model. (DOCX 15 kb) [file 12918_2018_668_MOESM3_ESM.docx]

function dsdt = coreFile_F_V(s,y,params)

s1 = y(1); % R

s2 = y(2); % F

s3 = y(3); % H

s4 = y(4); % FRS2

s5 = y(5); % MEK

s6 = y(6); % ERK

s7 = y(7); % ERKppN

s8 = y(8); % F:H

s9 = y(9); % F:R

s10 = y(10); % F:H:R

s11 = y(11); % F:H:R:R:H:F

s12 = y(12); % F:H:R:R:H:F_p

s13 = y(13); % F:H:R:R:H:F_i

s14 = y(14); % FRS2p

s15 = y(15); % MEKpp

s16 = y(16); % ERKpp:FRS2

s17 = y(17); % FRS2u

s18 = y(18); % ERKpp:FRS2p

s19 = y(19); % ERKpp

s20 = y(20); % FRS2:F:H:R:R:H:F_p

s21 = y(21); % FRS2p:MEK

s22 = y(22); % MEKpp:ERK

s23 = y(23); % pERK

s24 = y(24); % pERK:ppMEK

s25 = y(25); % pMEK

s26 = y(26); % FRS2p:pMEK

s27 = y(27); % Pase2

s28 = y(28); % Pase3

s29 = y(29); % ppMEK:Pase2

s30 = y(30); % pMEK:Pase2

s31 = y(31); % ppERK:Pase3

s32 = y(32); % pERK:Pase3

s33 = y(33); % R2

s34 = y(34); % iR2

s35 = y(35); % R2_p

s36 = y(36); % iR2_p

s37 = y(37); % [Ras-GDP]

s38 = y(38); % [R2_p:Ras-GDP]

s39 = y(39); % iR2_p

s40 = y(40); % [R2_p:Ras-GDP]

s41 = y(41); % [Ras-GTP]

s42 = y(42); % [aRas-GTP]

s43 = y(43); % [R2_p:Ras-GTP]

s44 = y(44); % [iR2_p:Ras-GTP]

s45 = y(45); % F:H:R:R:H:F_p_i

s46 = y(46); % PTP1B

s47 = y(47); % [R2_p:PTP1B]

s48 = y(48); % F:H_i

s49 = y(49); % F:R_i

s50 = y(50); % V

s51 = y(51); % Raf

s52 = y(52); % [Raf:Ras-GTP]

s53 = y(53); % aRaf

s54 = y(54); % [dR2_p:Ras-GDP]

s55 = y(55); % Pase1

s56 = y(56); % [Raf_a:Pase1]

s57 = y(57); % [MEK:Raf_a]

s58 = y(58); % [MEK_p:Raf_a]

s59 = y(59); % dR2

s60 = y(60); % dR2_p

s61 = y(61); % [dR2_p:Ras-GTP]

s62 = y(62); % F:H:R_i

s63 = y(63); % H_i

s64 = y(64); % R_i

s65 = y(65); % FRS2:F:H:R:R:H:F_i

s66 = y(66); % F:H_d

s67 = y(67); % F:R_d

s68 = y(68); % F:H:R_d

s69 = y(69); % H_d

s70 = y(70); % R_d

s71 = y(71); % FRS2:F:H:R:R:H:F_d

s72 = y(72); % F:H:R:R:H:F_d

kf0 = params( 1 ,1);

kr0 = params( 2 ,1);

kf1a = params( 3 ,1);

kr1a = params( 4 ,1);

kf5a = params( 5 ,1);

kr5a = params( 6 ,1);

kfdim = params( 7 ,1);

krdim = params( 8 ,1);

kfph = params( 9 ,1);

kfint1 = params( 10 ,1);

krint1 = params( 11 ,1);

kf15 = params( 12 ,1);

kr15 = params( 13 ,1);

kf19 = params( 14 ,1);

kf35 = params( 15 ,1);

kr35 = params( 16 ,1);

kf36 = params( 17 ,1);

kf37 = params( 18 ,1);

k_aERKMEK = params( 19 ,1);

kd_aERKMEK = params( 20 ,1);

ked_ERKMEKpp = params( 21 ,1);

ked_ERKpMEKpp = params( 22 ,1);

kfdp1 = params( 23 ,1);

ked2 = params( 24 ,1);

ked3 = params( 25 ,1);

kf43 = params( 26 ,1);

kr43 = params( 27 ,1);

kf44 = params( 28 ,1);

kf47 = params( 29 ,1);

kr47 = params( 30 ,1);

k_pR2 = params( 31 ,1);

kd_pR2 = params( 32 ,1);

k_aRafRasGTP = params( 33 ,1);

kd_aRafRasGTP = params( 34 ,1);

kd_RafRasGTP = params( 35 ,1);

kd_rRafRasGTP = params( 36 ,1);

k_dpRaf = params( 37 ,1);

kd_dpRaf = params( 38 ,1);

ked = params( 39 ,1);

k_aMEKRaf = params( 40 ,1);

kd_aMEKRaf = params( 41 ,1);

ked_MEKRaf = params( 42 ,1);

ked_MEKRaf2 = params( 43 ,1);

k_dpMEK_pp = params( 44 ,1);

kd_dpMEK_pp = params( 45 ,1);

k_dpERKpp = params( 46 ,1);

kd_dpERKpp = params( 47 ,1);

k_intf = params( 48 ,1);

k_recf = params( 49 ,1);

k_intb = params( 50 ,1);

k_recb = params( 51 ,1);

k_aRasGDP = params( 52 ,1);

kd_aRasGDP = params( 53 ,1);

k_degf = params( 54 ,1);

k_degb = params( 55 ,1);

kd_RasGDP = params( 56 ,1);

kd_rRasGDP = params( 57 ,1);

k_aRasGTP = params( 58 ,1);

kd_aRasGTP = params( 59 ,1);

kd_RasGTP = params( 60 ,1);

kd_rRasGTP = params( 61 ,1);

kd_v = params( 62 ,1);

k_ptp = params( 63 ,1);

kd_ptp = params( 64 ,1);

ke_ptp = params( 65 ,1);

k_dpMEK_p = params( 66 ,1);

kd_dpMEK_p = params( 67 ,1);

k_dpERKp = params( 68 ,1);

kd_dpERKp = params( 69 ,1);

kintb_f = params( 70 ,1);

krecb_f = params( 71 ,1);

kdegb_f = params( 72 ,1);

kdegf_f = params( 73 ,1);

kintf_f = params( 74 ,1);

krecf_f = params( 75 ,1);

R1 = kf0*s2*s3 - kr0*s8 ;

R2 = kf1a*s2*s1 - kr1a*s9 ;

R3 = kf5a*s1*s8 - kr5a*s10 ;

R4 = kfdim*s10*s10 - krdim*s11 ;

R5 = kfph*s11 ;

R6 = kfint1*s12 - krint1*s45 ;

R7 = kdegb_f*s13 ;

R8 = kf15*s4*s12 - kr15*s20 ;

R9 = kf19*s20 ;

R10 = kfdp1*s14 ;

R11 = kintb_f*s8 - krecb_f*s48 ;

R12 = kintb_f*s9 - krecb_f*s49 ;

R13 = kintb_f*s10 - krecb_f*s62 ;

R14 = kintb_f*s11 - krecb_f*s13 ;

R15 = kintf_f*s3 - krecf_f*s63 ;

R16 = kintf_f*s1 - krecf_f*s64 ;

R17 = kintb_f*s20 - krecb_f*s65 ;

R18 = kdegb_f*s48 ;

R19 = kdegb_f*s49 ;

R20 = kdegb_f*s62 ;

R21 = kdegb_f*s45 ;

R22 = kdegf_f*s63 ;

R23 = kdegf_f*s64 ;

R24 = kdegb_f*s65 ;

R25 = kf35*s14*s5 - kr35*s21 ;

R26 = kf36*s21 ;

R27 = kf35*s14*s25 - kr35*s26 ;

R28 = kf37*s26 ;

R29 = k_pR2*s50*s33-kd_pR2*s35 ;

R30 = k_aRafRasGTP*s51*s41-kd_aRafRasGTP*s52 ;

R31 = kd_RafRasGTP*s52-kd_rRafRasGTP*s53*s42 ;

R32 = k_dpRaf*s53*s55-kd_dpRaf*s56 ;

R33 = ked*s56 ;

R34 = k_aMEKRaf*s5*s53-kd_aMEKRaf*s57 ;

R35 = ked_MEKRaf*s57 ;

R36 = k_aMEKRaf*s25*s53-kd_aMEKRaf *s58 ;

R37 = ked_MEKRaf2*s58 ;

R38 = k_dpMEK_pp*s15*s27-kd_dpMEK_pp*s29 ;

R39 = ked2*s29 ;

R40 = k_dpMEK_p*s25*s27-kd_dpMEK_p*s30 ;

R41 = ked2*s30 ;

R42 = k_aERKMEK*s15*s6 - kd_aERKMEK*s22 ;

R43 = ked_ERKMEKpp*s22 ;

R44 = k_aERKMEK*s23*s15 - kd_aERKMEK*s24 ;

R45 = ked_ERKpMEKpp*s24 ;

R46 = k_dpERKpp*s19*s28-kd_dpERKpp*s31 ;

R47 = ked3*s31 ;

R48 = k_dpERKp*s23*s28-kd_dpERKp*s32 ;

R49 = ked3*s32 ;

R50 = kf43*s19*s4 - kr43*s16 ;

R51 = kf44*s16 ;

R52 = kf43*s19*s14 - kr43*s18 ;

R53 = kf44*s18 ;

R54 = kf47*s19 - kr47*s7 ;

R55 = k_intf*s33-k_recf*s34 ;

R56 = k_intb*s35-k_recb*s36 ;

R57 = k_aRasGDP*s35*s37-kd_aRasGDP*s38 ;

R58 = k_degf*s34 ;

R59 = k_degb*s36 ;

R60 = k_intb*s38-k_recb*s40 ;

R61 = kd_RasGDP*s38-kd_rRasGDP *s35*s41 ;

R62 = k_aRasGTP*s35*s42-kd_aRasGTP*s43 ;

R63 = kd_RasGTP*s43-kd_rRasGTP *s35*s37 ;

R64 = kd_v*s43 ;

R65 = kd_v*s38 ;

R66 = k_intb*s43-k_recb*s44 ;

R67 = k_degb*s44 ;

R68 = k_degb*s40 ;

R69 = k_ptp*s35*s46-kd_ptp*s47 ;

R70 = ke_ptp*s47 ;

dsdt( 1 ,1) = -R2-R3-R16 ;

dsdt( 2 ,1) = -R1-R2 ;

dsdt( 3 ,1) = -R1-R15 ;

dsdt( 4 ,1) = -R8+R10-R50 ;

dsdt( 5 ,1) = -R25-R34+R41 ;

dsdt( 6 ,1) = -R42+R49 ;

dsdt( 7 ,1) = R54 ;

dsdt( 8 ,1) = R1-R3-R11 ;

dsdt( 9 ,1) = R2-R12 ;

dsdt( 10 ,1) = R3-2*R4-R13 ;

dsdt( 11 ,1) = R4-R5-R14 ;

dsdt( 12 ,1) = R5-R6-R8+R9 ;

dsdt( 13 ,1) = -R7+R14 ;

dsdt( 14 ,1) = R9-R10-R25+R26-R27+R28-R52 ;

dsdt( 15 ,1) = R28+R37-R38-R42+R43-R44+R45 ;

dsdt( 16 ,1) = R50-R51 ;

dsdt( 17 ,1) = R51+R53 ;

dsdt( 18 ,1) = R52-R53 ;

dsdt( 19 ,1) = R45-R46-R50+R51-R52+R53-R54 ;

dsdt( 20 ,1) = R8-R9-R17 ;

dsdt( 21 ,1) = R25-R26 ;

dsdt( 22 ,1) = R42-R43 ;

dsdt( 23 ,1) = R43-R44+R47-R48 ;

dsdt( 24 ,1) = R44-R45 ;

dsdt( 25 ,1) = R26-R27+R35-R36+R39-R40 ;

dsdt( 26 ,1) = R27-R28 ;

dsdt( 27 ,1) = -R38+R39-R40+R41 ;

dsdt( 28 ,1) = -R46+R47-R48+R49 ;

dsdt( 29 ,1) = R38-R39 ;

dsdt( 30 ,1) = R40-R41 ;

dsdt( 31 ,1) = R46-R47 ;

dsdt( 32 ,1) = R48-R49 ;

dsdt( 33 ,1) = -R29-R55+R64+R65+R70 ;

dsdt( 34 ,1) = R55-R58 ;

dsdt( 35 ,1) = R29-R56-R57+R61-R62+R63-R69 ;

dsdt( 36 ,1) = R56-R59 ;

dsdt( 37 ,1) = -R57+R63+R64+R65 ;

dsdt( 38 ,1) = R57-R60-R61-R65 ;

dsdt( 39 ,1) = R21 ;

dsdt( 40 ,1) = R60-R68 ;

dsdt( 41 ,1) = -R30+R61 ;

dsdt( 42 ,1) = -R62+R31 ;

dsdt( 43 ,1) = R62-R63-R64-R66 ;

dsdt( 44 ,1) = R66-R67 ;

dsdt( 45 ,1) = R6-R21 ;

dsdt( 46 ,1) = -R69+R70 ;

dsdt( 47 ,1) = R69-R70 ;

dsdt( 48 ,1) = R11-R18 ;

dsdt( 49 ,1) = R12-R19 ;

dsdt( 50 ,1) = -R29+R64+R65 ;

dsdt( 51 ,1) = -R30+R33 ;

dsdt( 52 ,1) = R30-R31 ;

dsdt( 53 ,1) = R31-R32-R34+R35-R36+R37 ;

dsdt( 54 ,1) = R68 ;

dsdt( 55 ,1) = -R32+R33 ;

dsdt( 56 ,1) = R32-R33 ;

dsdt( 57 ,1) = R34-R35 ;

dsdt( 58 ,1) = R36-R37 ;

dsdt( 59 ,1) = R58 ;

dsdt( 60 ,1) = R59 ;

dsdt( 61 ,1) = R67 ;

dsdt( 62 ,1) = R13-R20 ;

dsdt( 63 ,1) = R15-R22 ;

dsdt( 64 ,1) = R16-R23 ;

dsdt( 65 ,1) = R17-R24 ;

dsdt( 66 ,1) = R18 ;

dsdt( 67 ,1) = R19 ;

dsdt( 68 ,1) = R20 ;

dsdt( 69 ,1) = R22 ;

dsdt( 70 ,1) = R23 ;

dsdt( 71 ,1) = R24 ;

dsdt( 72 ,1) = R7 ;

return
